# Quantum phases in twisted homobilayer transition metal dichalcogenides

**DOI:** 10.1093/nsr/nwaf570

**Published:** 2025-12-12

**Authors:** Bohao Li, Wen-Xuan Qiu, Fengcheng Wu, A H MacDonald

**Affiliations:** School of Physics and Technology, Wuhan University, Wuhan 430072, China; School of Physics and Technology, Wuhan University, Wuhan 430072, China; School of Physics and Technology, Wuhan University, Wuhan 430072, China; Wuhan Institute of Quantum Technology, Wuhan 430206, China; Department of Physics, University of Texas at Austin, Austin, Texas 78712, USA

**Keywords:** moiré materials, topological phases of matter, strongly correlated physics

## Abstract

Twisted homobilayer transition metal dichalcogenides—specifically twisted bilayer MoTe$_2$ and twisted bilayer WSe$_2$—have recently emerged as a versatile platform for strongly correlated and topological phases of matter. These two-dimensional systems host tunable flat Chern bands in which Coulomb interactions can dominate over kinetic energy, giving rise to a variety of interaction-driven phenomena. A series of groundbreaking experiments have revealed a rich landscape of quantum phases, including integer and fractional quantum anomalous Hall states, quantum spin Hall states, anomalous Hall metals, zero-field composite Fermi liquids and unconventional superconductors, along with more conventional topologically trivial correlated states, including antiferromagnets. This review surveys recent experimental discoveries and theoretical progress in understanding these phases, with a focus on the key underlying mechanisms—band topology, electron interactions, symmetry breaking and charge fractionalization. We emphasize the unique physics of twisted transition metal dichalcogenide homobilayers in comparison to other related systems, discuss open questions and outline promising directions for future research.

## Teaser

This article reviews quantum phases in semiconductor moiré superlattices, where topology and strong correlations drive a variety of phases including integer and fractional topological states, and superconductivity.

## INTRODUCTION

The field of moiré materials [1] has sparked a revolution in condensed matter physics by providing a new generation of quantum materials—one in which properties can be flexibly tuned by using different two-dimensional crystals with different lattice constant mismatches, by changing interlayer twists, and most crucially by changing gate voltages *in situ* to induce continuous property evolution. In semiconductor and semimetal hosts, moiré superlattices yield emergent periodic Hamiltonians—artificial large-lattice-constant crystals—with Bloch states and, frequently, flat electronic bands. The large periodicities allow gates to change the electron filling per moiré unit cell by ${\sim } 10$, effectively moving across the columns of a chemical periodic table. Each integer filling corresponds to a distinct entry in a *moiré periodic table* and intermediate filling factors correspond to disorder-free doping.

The emergence of moiré materials as an important topic in condensed matter physics was catalyzed by the discovery of correlated insulators and superconductivity in magic-angle twisted bilayer graphene (TBG) [2,3], but the field has since expanded to include a much broader range of moiré materials [4–7], including some based on transition metal dichalcogenides (TMDs). Among these, twisted TMD homobilayers—specifically twisted bilayer MoTe$_2$ (*t*MoTe$_2$) and twisted bilayer WSe$_2$ (*t*WSe$_2$) in the *R*-stacking configuration—have emerged as a particularly fertile ground for the discovery of new strongly correlated and topologically nontrivial phases of matter, including unusual examples of superconductivity. Monolayers of group-VIB TMDs, with chemical composition $MX_2$ ($M=$ Mo, W and *X* = S, Se, Te), are semiconductors in the *H* phase. In these monolayers, valence-band maxima are located at the two inequivalent corners of the hexagonal Brillouin zone, known as $\pm K$ valleys. Because of the absence of inversion symmetry, spin-orbit coupling generates a pronounced valley-contrasting Ising spin splitting in the valence bands, leading to an effective spin-valley locking [8]. When hole doping places the chemical potential near the valence-band maxima, the low-energy carriers can be modeled as massive particles possessing a valley pseudospin degree of freedom, which effectively behaves as the physical spin. This behavior is distinct from that of monolayer graphene, in which the low-energy carriers are described by massless Dirac fermions with both spin and valley degrees of freedom.

These intrinsic differences at the monolayer level result in qualitatively distinct physical properties in the twisted bilayer counterparts. In TMDs, spin-valley locking depletes the low-energy degrees of freedom, giving rise to moiré bands with a simplified flavor structure. The massive nature of low-energy carriers leads to moiré bands with narrow bandwidths over a broad range of twist angles, in sharp contrast to the magic-angle condition required to achieve flat bands in TBG. Furthermore, the lack of two-fold rotational symmetry about the out-of-plane ${z}$-axis in twisted homobilayer TMDs permits valley-contrasting Berry curvatures and enables the realization of topological moiré bands driven by interlayer hybridization. These characteristics are elegantly captured by a simple continuum moiré Hamiltonian [10], which reveals that particles move within a background layer pseudospin skyrmion texture, providing a real-space view of the emergence of moiré band topology. The top two moiré valence bands can carry opposite Chern numbers, realizing an effective Haldane model [11] in a single valley and a Kane–Mele model [12] when both valleys are considered.

Because of the narrow moiré bandwidths, electron interaction effects are significantly enhanced. Early theoretical studies proposed that at hole filling factor $\nu _h=1$ (one hole per moiré unit cell), the system could realize an integer quantum anomalous Hall state driven by spontaneous valley polarization [10]. Going beyond integer fillings, numerical studies based on exact diagonalization have further predicted fractional quantum anomalous Hall insulators at fractional hole fillings in *t*MoTe$_2$ [13]. The possibility of realizing such fractionalized states in twisted homobilayer TMDs was further supported by the theoretical observation that the moiré flat bands in this system can have relatively uniform Berry curvature distribution—a key condition for stabilizing fractional Chern insulators [14].

The potential of this platform was first revealed by a pioneering transport study of *t*WSe$_2$, which uncovered a topologically trivial correlated insulating state at $\nu _h=1$, indicating the strongly interacting nature of the system [15]. A major turning point in this field came with the discovery of both integer and fractional quantum anomalous Hall insulators in *t*MoTe$_2$ [16–19]. Although integer quantum anomalous Hall insulators had already been observed in various material platforms [20–32], *t*MoTe$_2$ was the first system to exhibit fractional quantum anomalous Hall effects at zero external magnetic field [18,19], marking a significant milestone in the pursuit of fractionalized topological phases.

Beyond these advances, twisted homobilayer TMDs have been shown to host a rich variety of quantum phases in a phase diagram tuned by the carrier density, displacement field and twist angle [15,18,33,34–37,38]. States discovered to date include integer and fractional quantum spin Hall insulating states [34,35], anomalous Hall metals [18], zero-field composite Fermi liquids [18,33], unconventional superconductors [36–38] and topologically trivial correlated phases such as intervalley antiferromagnets [15,37,38]. The emergence of these diverse quantum states has attracted broad interest and opened new research directions within the topological physics, superconductivity and quantum materials fields.

In this paper, we present a comprehensive review of the emergent quantum phases in twisted TMD homobilayers. We start by examining the moiré band structures, emphasizing their topological properties that lay the foundation for a deeper investigation of the resulting quantum phases. We then summarize key experimental findings, highlighting the experimental signatures of various quantum phases when examined with different probes. Following this, we explore in detail the integer and fractional topological states and the superconducting states, connecting them to theoretical frameworks that emphasize correlation effects within flat Chern bands. Throughout the review, we highlight the unique characteristics of twisted homobilayer TMDs in comparison to other related systems, and conclude with a discussion of open challenges and promising directions for future research.

## TOPOLOGICAL MOIRÉ BANDS

In twisted TMD homobilayers (both layers from the same material), configurations with twist angles $\theta$ near $0^\circ$ and $180^\circ$ are physically distinct. This is because the individual TMD monolayers lack $C_{2z}$ symmetry (i.e. two-fold rotation symmetry around the out-of-plane ${z}$-axis). These two cases correspond to different stacking types, referred to as *R* and *H*, respectively. The labels are inherited from terminology used to describe the corresponding untwisted bilayers: *R* (rhombohedral) refers to the aligned configuration and *H* (hexagonal) to the antialigned configuration. In the *H* stacking of twisted bilayers, opposite layers have opposite spins and time-reversed atomic-orbital wave functions in a given valley. The spin mismatch suppresses interlayer tunneling, effectively decoupling the two layers at low energies at the single-particle level. Consequently, *H* stacking can realize a bilayer generalized Hubbard model in which layer and valley degrees of freedom together give rise to an approximate SU(4) symmetry in a valley/layer flavor space [10,39–41]. In contrast, in *R* stacking momentum-aligned valleys in the two layers carry the same spin and have the same atomic orbital, allowing for stronger interlayer tunneling that imposes qualitatively different physics.

It is important to note that the location of the valence-band maximum in twisted TMD homobilayers can vary depending on the material. For example, the valence-band maxima shift to the Brillouin zone center (i.e. the $\Gamma$ valley [42]) in twisted bilayers of *t*MoS$_2$, *t*WS$_2$ and *t*MoSe$_2$, but remain in the $\pm K$ valleys in *t*WSe$_2$ and *t*MoTe$_2$. These two cases have qualitatively distinct electronic structure and low-energy physics. In this review article, we focus on *t*MoTe$_2$ and *t*WSe$_2$ in the *R*-stacking configuration, in which the low-energy physics is controlled by $\pm K$ valley band states with strong interlayer hybridization.

**Figure 1.**
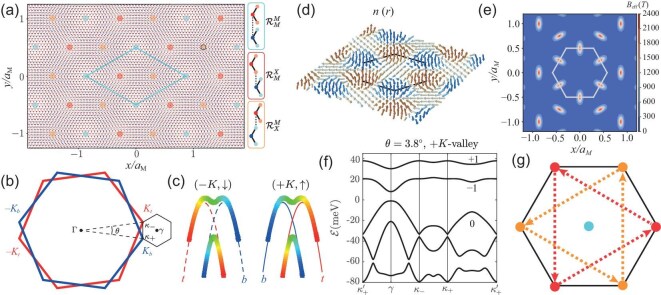
Single-particle physics in twisted homobilayer TMDs. (a) Moiré superlattices of *R*-stacked twisted homobilayers. The dots identify the high-symmetry positions $\mathcal {R}_M^M$, $\mathcal {R}_X^M$ and $\mathcal {R}_M^X$, and the solid lines outline a single moiré unit cell. (b) Brillouin zones of the bottom (blue) and top (red) layers in a twisted bilayer, and the moiré Brillouin zone (black). (c) Moiré band states near the valence-band maxima of two valleys. The color shading indicates the degree of layer polarization of the corresponding layer spinors (bottom, blue; top, red). (d) Map of the skyrmion field $n(\boldsymbol{r})$ and (e) the corresponding effective magnetic field $B_{\mathrm{eff}}(\boldsymbol{r})$ of *t*MoTe$_2$ at $\theta = 3.8^\circ$. The black hexagon in (d) (the white hexagon in (e)) is the Wigner–Seitz cell of the moiré superlattice. (f) Moiré band structure of *t*MoTe$_2$ at $\theta = 3.8^\circ$ calculated from the continuum model. The numbers $(+1,-1,0)$ are the Chern numbers of the first three moiré valence bands. (g) The honeycomb lattice formed by $\mathcal {R}_X^M$ and $\mathcal {R}_M^X$ sites. Red and orange dashed arrows indicate the phase pattern of next-nearest-neighbor hopping in the effective Haldane model for the first two bands in (f). Panels (a–c) are adapted from [9].

The moiré superlattices formed in an *R*-stacked twisted homobilayer is illustrated in Fig. 1a. For small $\theta$, the superlattices have a moiré period $a_M \approx a_0/\theta$, where $a_0$ is the in-plane constant of the monolayer and $\theta$ is the twist angle in radians. The twisted bilayer possesses $D_3$ point-group symmetry generated by a three-fold rotation $C_{3z}$ around the ${z}$-axis and a two-fold rotation $C_{2y}$ around the in-plane ${y}$-axis that exchanges the two layers. Within a moiré unit cell, there are three positions invariant under $C_{3z}$: $\mathcal {R}_{M}^M$, $\mathcal {R}_{M}^X$ and $\mathcal {R}_{X}^M$. Here $\mathcal {R}_{\alpha }^{\beta }$ denotes the local atomic registry where the $\alpha$ atom in the bottom TMD layer is vertically aligned with the $\beta$ atom in the top layer. Under the $C_{2y}$ operation, the $\mathcal {R}_{M}^M$ sites remain invariant, while the $\mathcal {R}_{M}^X$ and $\mathcal {R}_{X}^M$ sites are exchanged.

In momentum space, states in $+K$ and $-K$ valleys can be treated separately in the single-particle Hamiltonian, since they are separated by a large momentum when $\theta$ is small (Fig. 1b). The two valleys are related by the $C_{2y}$ symmetry and by time-reversal symmetry $\mathcal {T}$. For definiteness, we describe the single-particle physics in the $+K$ valley, with the understanding that the $-K$ valley states can be obtained via the action of $C_{2y}$ or $\mathcal {T}$ symmetries. The low-energy valence states in the $+K$ valley carry spin up ($\uparrow$) due to spin-valley locking and are described by the moiré Hamiltonian [10]


(1) \begin{eqnarray*}
&&\mathcal {H}_\uparrow\\ &&= \left({\begin{array}{cc}
-\frac{\hbar ^2 (\hat{\boldsymbol {k}}-\boldsymbol {\kappa }_+)^2}{2m^{*}}+\Delta _{+}(\boldsymbol {r}) &\,\, \Delta _{\mathrm{T}}(\boldsymbol {r}) \\
\Delta _{\mathrm{T}}^\dagger (\boldsymbol {r}) &\!\!\!\!\!\!\! -\frac{\hbar ^2(\hat{\boldsymbol {k}}-\boldsymbol {\kappa }_-)^2}{2m^{*}}
+\Delta _{-}(\boldsymbol {r})
\end{array}}\right)\!\!,\\
\end{eqnarray*}

where the $2\times 2$ matrix is in the layer pseudospin space, the diagonal terms are intralayer Hamiltonians and the off-diagonal terms describe the interlayer tunneling. Here, $\boldsymbol r$ and $\hbar \hat{\boldsymbol k}$ are respectively the position and momentum operators, and $m^{*}$ is the valence-band effective mass. The layer-dependent momentum offsets $\boldsymbol {\kappa }_{\pm }=[4\pi /(3a_M)](-\sqrt{3}/2,\mp 1/2)$ capture the relative momentum shift of valence-band maxima between the two twisted layers (Fig. 1c). The layer-dependent moiré potential $\Delta _{\pm }(\boldsymbol {r})$ is given by


(2) \begin{eqnarray*}
\Delta _{\pm }(\boldsymbol {r}) = 2 V \sum _{j=1,3,5}^{}\cos (\boldsymbol {g}_j\cdot \boldsymbol {r} \pm \psi ),
\end{eqnarray*}

where *V* and $\psi$ respectively characterize the amplitude and spatial pattern of the moiré potential. The interlayer tunneling $\Delta _{\mathrm{T}}(\boldsymbol {r})$ is fully characterized by a strength parameter *w*:


(3) \begin{eqnarray*}
\Delta _{\mathrm{T}}(\boldsymbol {r}) = w (1+e^{-i \boldsymbol {g}_2 \cdot \boldsymbol {r}}+e^{-i \boldsymbol {g}_3 \cdot \boldsymbol {r}}).
\end{eqnarray*}

In Equations (2) and (3), $\boldsymbol{g}_{j}=\frac{4\pi }{\sqrt{3}a_M} [\cos \frac{\pi (j-1)}{3},\sin \frac{\pi (j-1)}{3}]$are moiré reciprocal lattice vectors in the first momentum shell.

The continuum Hamiltonian $\mathcal {H}_\uparrow$ acts on envelope functions that vary on the moiré length scale and assumes that interlayer tunneling is accurately local on that scale. Moiré translation symmetry with period $a_M$ justifies the reciprocal lattice Fourier expansions of $\Delta _{\pm }$ and $\Delta _{T}$, and the large ratio of interlayer to intralayer atomic distances justifies the blue truncation at the leading harmonics. The number of independent model parameters is further limited by applying $C_{3z}$ and $C_{2y} \mathcal {T}$ symmetries. Because of the combined $C_{2y} \mathcal {T}$ symmetry, $\Delta _{+}(x,y)=\Delta _{-}(-x,y)$. The interlayer tunneling term $\Delta _{\mathrm{T}}(\boldsymbol {r})$ has zeros at the $\mathcal {R}_{M}^X$ and $\mathcal {R}_{X}^M$ positions that are protected by the $C_{3z}$ symmetry. An important feature of $\Delta _{\mathrm{T}}(\boldsymbol {r})$ is its complex spatial dependence on $\boldsymbol {r}$, which originates from the spatially varying phase factors of the Bloch states at the Brillouin zone corners. We note that $\mathcal {H}_\uparrow$ in Equation (1) includes only quadratic kinetic energy terms and lowest-harmonic expansions in the potential terms. While higher-order terms are allowed and can be added when there is a motivation, $\mathcal {H}_\uparrow$ succinctly captures the essential physics.

The symmetry properties of $\Delta _{\pm }(\boldsymbol r)$ and $\Delta _{\mathrm{T}}(\boldsymbol r)$ imply a skyrmion lattice in real space, which is revealed by decomposing the moiré Hamiltonian using layer pseudospin Pauli matrices [10]. A unitary transformation can be applied to $\mathcal {H}_\uparrow$ to make its form more symmetric,


(4) \begin{eqnarray*}
\mathcal {H}_{\uparrow }^{^{\prime }} &=& U_{0}^\dagger (\boldsymbol{r})\mathcal {H}_{\uparrow }U_{0}(\boldsymbol{r}), \\
&=& -\frac{\hbar ^2\hat{\boldsymbol{k}}^2}{2m^{*}}\sigma _0+\boldsymbol{\Delta }(\boldsymbol{r})\cdot \boldsymbol{\sigma }+\Delta _0(\boldsymbol{r})\, \sigma _0,
\end{eqnarray*}

where $U_0(\boldsymbol r)=\mathrm{diag}(e^{i\boldsymbol{\kappa }_+\cdot \boldsymbol{r}},e^{i\boldsymbol{\kappa }_-\cdot \boldsymbol{r}})$, $\sigma _0$ is the identity matrix and $\boldsymbol{\sigma }$ are Pauli matrices. The scalar potential $\Delta _0(\boldsymbol{r})$ and the layer pseudospin field $\boldsymbol{\Delta }(\boldsymbol{r})$ are defined as


(5) \begin{eqnarray*}
{\begin{array}{c}\Delta _0(\boldsymbol{r})= \displaystyle\frac{\Delta _+(\boldsymbol{r})+\Delta _-(\boldsymbol{r})}{2}, \\
\boldsymbol{\Delta }(\boldsymbol{r})\!\!=\!\! \bigg [\!\mathrm{Re}\, \widetilde{\Delta }_{\mathrm{T}}^\dagger (\boldsymbol{r}),\mathrm{Im}\, \widetilde{\Delta }_{\mathrm{T}}^\dagger (\boldsymbol{r}), \displaystyle\frac{\Delta _+(\boldsymbol{r})-\Delta _-(\boldsymbol{r})}{2}\!\bigg ], \\
\end{array}}\!\!\!\!\!\\
\end{eqnarray*}

where


(6) \begin{eqnarray*}
\widetilde{\Delta }_{\mathrm{T}}^\dagger (\boldsymbol{r})=e^{i(\boldsymbol{\kappa }_+-\boldsymbol{\kappa }_-)\cdot \boldsymbol{r}}\Delta _{\mathrm{T}}^\dagger (\boldsymbol{r}).
\end{eqnarray*}

The key observation is that the tunneling components of $\boldsymbol{\Delta }$ have vortex and antivortex textures around the $\mathcal {R}_{M}^{X}$ and $\mathcal {R}_{X}^{M}$ positions, while the *z* component $\Delta _z$ takes opposite values at these two high-symmetry positions. This spatial profile indicates that $\boldsymbol {\Delta }$ forms a skyrmion texture, characterized by the winding number $N_w$,


(7) \begin{eqnarray*}
N_w &\equiv& \frac{1}{4\pi }\int _{\mathrm{MUC}}d\boldsymbol {r} \,\, [\boldsymbol {n}\cdot (\partial _x \boldsymbol {n} \times \partial _y \boldsymbol {n})] \\
&=&\left\lbrace \begin{array}{@{}l@{\quad }l@{}}+1,& V\sin \psi >0,\\
-1,& V\sin \psi <0, \end{array}\right.
\end{eqnarray*}

where the integral is over the moiré unit cell (MUC). Here $\boldsymbol {n}$ denotes a unit vector in the direction of $\boldsymbol {\Delta }$. When $V\sin \psi > 0$, $\boldsymbol {n}$ points toward the north (south) pole at $\mathcal {R}_{M}^X$ ($\mathcal {R}_{X}^M$). This orientation reverses when $V\sin \psi < 0$, implying a change in sign of the winding number $N_w$.

In the adiabatic limit where the particle’s pseudospin follows the skyrmion texture locally, there is an emergent orbital magnetic field $B_{\mathrm{eff}}$ [9,10,43–45]:


(8) \begin{eqnarray*}
B_{\mathrm{eff}}(\boldsymbol {r})=\frac{\hbar }{2e} \boldsymbol {n}\cdot (\partial _x \boldsymbol {n} \times \partial _y \boldsymbol {n}).
\end{eqnarray*}

The effective magnetic flux produced by $B_{\mathrm{eff}}$ over one MUC is quantized to $\pm \Phi _0$, where $\Phi _0=h/e$ is the magnetic flux quantum, following Equation (7). The spatial-averaged value of $B_{\mathrm{eff}}$ has a magnitude of $\bar{B}=h/(e\mathcal {A}_0)$, where $\mathcal {A}_0=\sqrt{3}a_M^2/2$ is the area of one MUC. We can further define an effective magnetic length as


(9) \begin{eqnarray*}
\ell _0=\sqrt{\frac{\hbar }{e\bar{B}}}=\sqrt{\frac{\sqrt{3}}{4\pi }}a_M\approx 0.37 a_M.
\end{eqnarray*}

For a typical moiré period $a_M$ of 5 nm, $\ell _0 \approx 1.85$ nm and $\bar{B} \approx 191$ T—more than an order of magnitude larger than magnetic fields typically accessible in laboratory settings. Note that $\bar{B}$ scales inversely with the square of the moiré period, i.e. $\bar{B} \propto 1/a_M^2 \propto \theta ^2$.

The skyrmion lattice and the effective magnetic field suggest the possibility of topological moiré bands for $\mathcal {H}_{\uparrow }$. However, even in the adiabatic limit of long moiré periods, the quantum particles experience not only the $B_{\mathrm{eff}}$ field but also the scalar potential $\Delta _0(\boldsymbol r)+|\boldsymbol \Delta (\boldsymbol r)|-D(\boldsymbol r)$. Here $D(\boldsymbol r)=(\hbar ^2 / 8 m^{*}) \sum _{i=x, y}[\partial _i \boldsymbol{n}]^2$, which arises from the precession of the layer pseudospin [9,43–45]. Because of the interplay among different terms, the topology of moiré bands depends on the model details. A systematic investigation of band topology as a function of the model parameters can be found in [9]. A rule of thumb is that the topmost moiré valence band has a nonzero Chern number if the low-energy particles are confined to one layer at $\mathcal {R}_{M}^{X}$ positions and to the other at $\mathcal {R}_{X}^{M}$ positions, forming an effective buckled honeycomb lattice [9].

The model parameters can be obtained from first-principles band-structure calculations, which have been carried out for moiré superlattices using large-scale density-functional-theory (DFT) simulations [14,46–52]. Although the predictions of different studies differ in detail, they consistently indicate that the low-energy moiré valence bands in both *t*MoTe$_2$ and *t*WSe$_2$ are topological. A representative set of parameters, extracted by fitting the DFT band structure of *t*MoTe$_2$ at a twist angle $\theta = 3.89^\circ$ is as follows: effective mass $m^{*} \approx 0.6 m_e$, potential amplitude $V = 20.8$ meV, phase $\psi = 107.7^\circ$ and interlayer tunneling strength $w = -23.8$ meV, where $m_e$ is the electron bare mass [47]. The monolayer MoTe$_2$ lattice constant is $a_0 = 3.52$ Å. The skyrmion field $\boldsymbol {n}(\boldsymbol {r})$ in Fig. 1d and the corresponding effective magnetic field $B_{\mathrm{eff}}(\boldsymbol {r})$ in Fig. 1e are calculated using these parameters with $\theta = 3.8^\circ$. The $B_{\mathrm{eff}}(\boldsymbol {r})$ field has strong spatial variations, reaching peak values of 2400 T for $\theta = 3.8^\circ$—an order of magnitude larger than the average value of 170 T—at the three points in each unit cell that are midway between the $\mathcal {R}_{M}^{X}$ and $\mathcal {R}_{X}^{M}$ points.

The moiré band structure with $\theta = 3.8^\circ$ is shown in Fig. 1f. It is calculated using Hamiltonian $\mathcal {H}_{\uparrow }$ with the parameters listed above for *t*MoTe$_2$. The first (topmost) moiré valence band is energetically isolated from other bands, has a narrow bandwidth of about 8 meV and carries a finite Chern number of $C_{+K,1}=+1$. Here we use $C_{\pm K,n}$ to denote the Chern number of the *n*th moiré band in the $\pm K$ valley. Because of time-reversal symmetry $\mathcal {T}$, the Chern numbers at opposite valleys are related by $C_{-K,n}=-C_{+K,n}$. In Fig. 1f, the Chern numbers for the second and third bands are respectively $-1$ and 0. In this case, the total Chern number of the first two bands is zero, allowing a tight-binding description in terms of two Wannier states, which are polarized to opposite layers and localized, respectively, at the $\mathcal {R}_{M}^{X}$ and $\mathcal {R}_{X}^{M}$ positions [10,14,53]. As illustrated in Fig. 1g, this tight-binding model, defined on an effective honeycomb lattice, includes complex next-nearest-neighbor hopping with the same pattern as in the Haldane model, reproducing its nontrivial band topology. Therefore, the first two bands realize the Haldane model [11] within a single valley and effectively implement the Kane–Mele model (i.e. two time-reversal partners of the Haldane model) [12] when both valleys are taken into account. The third moiré band is topologically trivial and can be described by a tight-binding model based on one Wannier orbital localized at the $\mathcal {R}_{M}^{M}$ position [53].

The Chern numbers of the moiré bands vary with the twist angle $\theta$, reflecting the explicit $\theta$ dependence of the moiré Hamiltonian through the moiré period. In addition, the model parameters $(V, \psi , w)$ also evolve with $\theta$ because of lattice relaxation effects. Large-scale DFT calculations for *t*MoTe$_2$ in [50,52] reveal the following behavior:


(10) \begin{eqnarray*}
&(C_{+K,1}, C_{+K,2}, C_{+K,3}) \\
&= \left\lbrace \begin{array}{@{}l@{\quad }l@{}}(1, 1, 1), &\quad \theta = 2.13^\circ , \\
(1, 1, -2), &\quad 2.45^\circ \le \theta \le 2.88^\circ , \\
(1, -1, 0), &\quad 3.15^\circ \le \theta \le 3.89^\circ . \end{array}\right.
\end{eqnarray*}

Remarkably, at $\theta = 2.13^\circ$, the first three moiré bands carry the same Chern number of $+1$, closely resembling the sequence of Landau levels induced by an external magnetic field. However, the critical twist angles at which the Chern numbers change can vary depending on the van der Waals functionals used in DFT studies [49,54], highlighting the sensitivity of the quantitative results to the DFT calculation details.

Similar topological physics emerges in *t*WSe$_2$. Large-scale DFT calculations in [52] show that, for $2.13^\circ \le \theta \le 3.89^\circ$, the Chern numbers of the first three $+K$ valley moiré valence bands in *t*WSe$_2$ are $(C_{+K,1}, C_{+K,2}, C_{+K,3}) = (+1, +1, -1)$. Another study [50] reports that $(C_{+K,1}, C_{+K,2})$ flips to $(-1,-1)$ in *t*WSe$_2$ when $\theta$ decreases to $1.25^{\circ }$. This transition arises from a reversal of the local layer polarization at $\mathcal {R}_M^{X}$ and $\mathcal {R}_X^{M}$ stacking points, driven by a competition between moiré ferroelectricity and piezoelectric effects [50]. At a given twist angle the moiré bands in *t*WSe$_2$ generally exhibit larger bandwidths than those in *t*MoTe$_2$, which can be attributed to the smaller effective mass $m^{*}$ in *t*WSe$_2$.

In addition to twist angle, the out-of-plane electric displacement field provides another effective tuning knob to control band topology. The displacement field breaks the layer degeneracy by generating a potential difference between the two layers. As a result, the layer polarization and Berry curvature distribution of the moiré bands can be altered. Topological phase transitions occur at critical displacement field values at which the Chern numbers of specific bands change discontinuously. Such field-induced transitions can be demonstrated using the continuum moiré Hamiltonian, which lays the foundation for electrically controllable topological states [10].

The presence of narrow moiré bandwidths significantly enhances the importance of Coulomb interactions relative to kinetic energy, often leading to interaction-driven ground states that go beyond single-particle physics. For example, the bandwidth of *t*MoTe$_2$ remains on the order of 10 meV for $\theta$ ranging from $2^{\circ }$ to $4^{\circ }$ [52], corresponding to a moiré period $a_M$ between 10 and 5 nm. The characteristic energy scale of Coulomb interactions is $e^2/(\epsilon a_M)$, which varies from 14 to 28 meV over the same $a_M$ range, assuming a dielectric constant $\epsilon = 10$. This interaction energy scale is comparable to, or even larger than, the single-particle bandwidth of the topmost moiré valence band, placing the system in the moderate to strongly correlated regime [10,14,47,52]. The interplay between electron correlations and nontrivial band topology—together with valley (spin), layer, and orbital degrees of freedom—leads to a rich landscape of quantum phases that we now discuss.

## OVERVIEW OF QUANTUM PHASES

**Figure 2.**
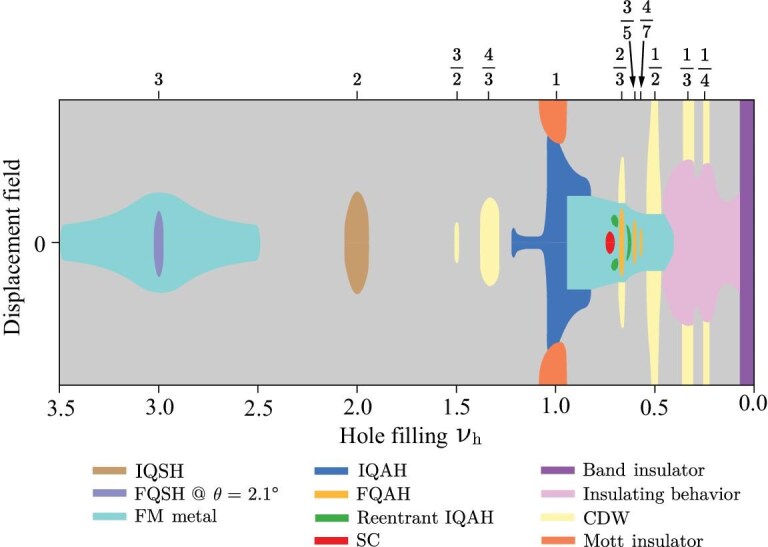
Schematic phase diagram of *t*MoTe$_2$ as a function of the hole filling $\nu _h$ and displacement field. This phase diagram summarizes experimental observations in [16–19,34–36,55,56] across twist angles ranging from $2.1^{\circ }$ to $3.9^{\circ }$. Some of the phases appear only at certain devices and twist angles. For example, the superconductor (SC) around $\nu _h \approx 0.73$ has so far been reported only in a device of *t*MoTe$_2$ with $\theta =3.83^{\circ }$ [36]. Evidence of an FQSH insulator has been reported in *t*MoTe$_2$ with $\theta =2.1^{\circ }$ [34]. FM metal refers to a ferromagnetic metal.

Recent experimental breakthroughs have revealed a wide variety of quantum phases in *t*MoTe$_2$ and *t*WSe$_2$, including, for example, integer and fractionalized topological states, as well as superconductivity. We begin by providing an overview of the available experiments, starting with *t*MoTe$_2$. In Fig. 2, we present a schematic phase diagram versus hole filling factor $\nu _h$ and out-of-plane displacement field. The phase diagram is based on the experimental observations in [16–19,34–36,55,56]. Here $\nu _h$ denotes the number of doped holes per moiré cell. At $\nu _h = 0$, the system is a bulk intrinsic *t*MoTe$_2$ semiconductor without carrier doping and is therefore a band insulator.

An integer quantum anomalous Hall (IQAH) insulator has been observed at $\nu _h = 1$ around zero displacement field over a broad range of twist angles $\theta$ from $2.1^\circ$ to $3.9^\circ$ [18,19,35,36,56]. This phase is characterized by a Hall resistance $R_{xy}$ with magnitude quantized to $h/e^2$ and a vanishing longitudinal resistance $R_{xx}$ at zero external magnetic field. In the IQAH state at $\nu _h = 1$, holes fully occupy the topmost moiré valence band in a single valley, as a result of flat-band ferromagnetism driven by Coulomb interactions. Spontaneous valley polarization along with nontrivial band topology explains a IQAH phase that breaks time-reversal symmetry [53,57]. The emergence of ferromagnetism for the magnetic insulator at small displacement fields, instead of the antiferromagnetism common in Hubbard-model systems, is related to the topologically nontrivial nature of the flat band. The IQAH insulator remains stable up to a critical displacement field, beyond which it undergoes a phase transition to a topologically trivial Mott insulator with carriers polarized to a single layer.

Fractional quantum anomalous Hall (FQAH) insulators emerge at fractional fillings $\nu _h=2/3, 3/5$ and $4/7$, displaying vanishing longitudinal resistances $R_{xx}$ and Hall resistances $R_{xy}$ with magnitudes quantized to $h/(\nu _h e^2)$ [18,19,35,36,56]. Like the IQAH insulator, the FQAH insulators have spontaneous spin/valley ferromagnetism persist up to critical displacement fields, after which they undergo a phase transition to a charge density wave (CDW) state at $\nu _h = 2/3$ or metallic states at $\nu _h = 3/5$ and $\nu _h = 4/7$.

In both IQAH and FQAH insulators, the Hall resistances $R_{xy}$ exhibit a hysteresis loop and switch sign when the out-of-plane magnetic field *B* is swept around zero, as is typical for spontaneous ferromagnetism [18,19,35,36,56]. Furthermore, the carrier density *n* in the IQAH and FQAH insulators varies linearly with the *B* field, as described by the Streda formula [58]:


(11) \begin{eqnarray*}
\sigma _{xy}=-e \frac{\partial n}{\partial B}=-\mathcal {C}\frac{e^2}{h}.
\end{eqnarray*}

Here $\sigma _{xy}$ is the Hall conductivity, *e* is the elementary charge and $\mathcal {C}$ is the Chern number of the many-body system. In these IQAH and FQAH insulators, $|\mathcal {C}|$ is quantized to the corresponding $\nu _h$ at zero magnetic field. In the Streda formula, the derivative $\partial n/\partial B$ is taken within the gap of the IQAH (or FQAH) insulators in theory, and along the trajectory with minimal (ideally vanishing) longitudinal resistance $R_{xx}$ in experiment.

The IQAH and FQAH insulators in *t*MoTe$_2$ have been characterized by several experimental probes in addition to transport. Optical measurements have played a key role because they sidestep the difficulty of making devices with Ohmic electrical contacts to the TMD bilayers. Reflective magnetic circular dichroism (RMCD) measurements [16,17,55] can identify ferromagnetism, as evidenced by remnant RMCD signals at zero external field for $\nu _h \in (0.4,1.2)$. Photoluminescence (PL) spectra have been employed as topological state sensors [16]. In particular, the spectrally integrated PL intensity has dips around hole fillings of $\nu _h=1, 2/3$ and $3/5$, and is attributed to reduced trion populations when the formation of correlated insulating states depletes the holes available for trion formation. Moreover, the dips show linear shifts in carrier density with an applied magnetic field that match the Streda formula in Equation (10) for the corresponding $\nu _h$. These optical properties provided evidence for IQAH and FQAH insulators in *t*MoTe$_2$ prior to transport studies, which require more stringent device fabrication procedures. Similar evidence was also provided by local electronic compressibility measurements, in which the states at $\nu _h=1$ and $2/3$ were found to be incompressible and to shift in density with an applied magnetic field, again consistent with the corresponding Streda formula [17].

The magnetization of *t*MoTe$_2$ has been further characterized using nanoscale superconducting quantum interference devices, which map the magnetic fringe fields [59]. A magnetic signal has been observed over a range of carrier densities and displacement fields, consistent with both optical and transport measurements. Magnetization jumps, reconstructed from the measured magnetic fields, appear at $\nu _h=1$ and $2/3$ that arise from the topological magnetization contributed by equilibrium chiral edge states. The change in magnetization $\delta m$ across the incompressible bulk gap is theoretically given by


(12) \begin{eqnarray*}
\delta m = \mathcal {C} \Delta _g / \Phi _0,
\end{eqnarray*}

where $\mathcal {C}$ is the Chern number of the system, $\Delta _g$ is the thermodynamic energy gap and $\Phi _0=h/e$. Equation (12) is related to the Streda formula since


(13) \begin{eqnarray*}
\frac{\partial m}{\partial \mu }\bigg |_B = \frac{\partial n}{\partial B}\bigg |_\mu = \frac{\mathcal {C}}{\Phi _0}.
\end{eqnarray*}

In Equation (13), the first equality follows from a Maxwell relation [60] and the second from the Streda formula in Equation (11). As the chemical potential $\mu$ varies across the bulk energy gap, it changes by $\Delta _g$, resulting in the magnetization change in Equation (12). The measured $\delta m$ has been used to estimate $\Delta _g$, yielding values of approximately 14 meV at $\nu _h=1$ and 7 meV at $\nu _h=2/3$ in a device with a twist angle $\theta =3.64^{\circ }$ [59].

Real-space imaging of quantum states in *t*MoTe$_2$ has also been performed using scanning microwave impedance microscopy with sub-100-nm spatial resolution [61]. This technique has visualized insulating bulk states and conductive edge states in the IQAH insulator at $\nu _h=1$ and the FQAH insulators at $\nu _h=2/3$ and $3/5$. These observations are consistent with the bulk-boundary correspondence of the topological states and confirm that quantized anomalous Hall conductances are associated with conductive edges.

Beyond the IQAH and FQAH insulators, *t*MoTe$_2$ hosts a rich variety of other phases, as shown in Fig. 2. The insulating regime at low fillings $\nu _h \lesssim 0.4$ centered on zero displacement field (pink region in Fig. 2) is insulating in current devices, possibly due to charge localization from disorder and/or interactions [18,19,35,36,56]. Contact issues may also contribute to the observed properties in this low-density regime. Over this range of $\nu _h$, the system becomes conductive above a filling-dependent critical displacement field, except at $\nu _h = 1/3$ and $1/4$ [36]. The latter two insulating states can be attributed to the interaction-driven CDW phases expected in triangular-lattice extended Hubbard models at strong interactions and commensurate fillings, and realized in the layer-polarized regime under high displacement fields.

Bulk states become metallic for $0.4 \lesssim \nu _h < 1$, except at certain fillings, such as $\nu _h = 2/3$, $3/5$ and $4/7$, where FQAH insulators emerge. The metallic states exhibit anomalous Hall effects around zero displacement field (cyan region in Fig. 2), indicating itinerant electron ferromagnetism from spontaneous valley polarization [18]. The anomalous Hall metal at $\nu _h=1/2$ has attracted particular attention. Near this filling, the Hall resistance $R_{xy}$ varies linearly with $\nu _h$ and crosses $2e^2/h$ at $\nu _h=1/2$, while the longitudinal resistance $R_{xx}$ remains at a few kilohms, consistent with a compressible state [18]. This behavior resembles that of the composite Fermi liquid near half-filling of the lowest Landau level in high magnetic fields [62–67], consistent with numerical predictions of a zero-field composite Fermi liquid at $\nu _h = 1/2$ in *t*MoTe$_2$ [68,69]. Optical signatures of this zero-field composite Fermi liquid have also been reported [33].

A recent transport study performed on a $\theta =3.83^{\circ }$ sample with reduced disorder and enhanced mobility uncovered additional exotic states at $\nu _h < 1$ [36]. A reentrant IQAH insulator emerges between the $\nu _h = 2/3$ and $3/5$ FQAH insulators near zero displacement field [36]. This phase exhibits a Hall resistance $R_{xy}$ quantized to $h/e^2$ and vanishing $R_{xx}$—hallmarks of the IQAH effect—yet occurs at an incommensurate filling $\nu _h \approx 0.63$ that is not associated with a simple rational fraction. This reentrant IQAH state is reminiscent of the reentrant integer quantum Hall effect seen in Landau levels with short-range disorder [71], and is likely an anomalous Hall crystal with spontaneous translational symmetry breaking [72]. Another reentrant IQAH insulator appears at $\nu _h \approx 0.7$, but only at a finite displacement field.

Remarkably, signatures of superconductivity have been observed in the same device around zero displacement field for $\nu _h \in (0.71,0.76)$. The superconducting state is separated from the $\nu _h=2/3$ FQAH insulator and the reentrant IQAH insulator by a resistance peak [36]. The superconducting zero-resistance state is reached up to 300 mK, with a perpendicular critical magnetic field as high as approximately 0.6 T. Above the superconducting transition temperature, the system enters a metallic phase with an anomalous Hall effect and magnetic hysteresis, indicating that the superconductor likely emerges from a ferromagnetic parent state. The embedding of a superconducting phase within a ferromagnetic metal is quite unusual. Moreover, the proximity of this state to the FQAH and reentrant IQAH insulators highlights its unconventional nature and calls for further experimental and theoretical exploration.

For $\nu _h > 1$, correlated insulators have been observed at $\nu _h = 3/2$ and $4/3$, likely corresponding to CDW states stabilized by electron-electron interactions [36]. At $\nu _h = 2$, multiple signatures point to an integer quantum spin Hall (IQSH) insulator around zero displacement field [34,35], including (1) vanishing Hall resistance and the absence of a remnant RMCD signal, (2) quantized two-terminal resistance plateaus at $h/2e^2$, (3) nonlocal transport behavior and (4) strong magnetoresistance under a small in-plane magnetic field combined with weak response to an out-of-plane field. These observations suggest that the $\nu _h = 2$ state is nonmagnetic and realizes the quantum spin Hall insulator expected [10] in the absence of interactions. In this state the topmost moiré valence bands from the $\pm K$ valleys—characterized by opposite out-of-plane spins $S_z$ and opposite Chern numbers—are fully hole doped. Helical edge states have opposite velocities for opposite $S_z$. In TMD homobilayer moiré materials the Ising $S_z$ symmetry provides additional protection for the helical edge states on top of that afforded by time-reversal symmetry. An in-plane (out-of-plane) magnetic field breaks (preserves) the $S_z$ symmetry, explaining the magnetoresistance behavior.

For $2<\nu _h<4$, the second moiré valence bands of the $\pm K$ valleys are partially filled. Ferromagnetism has been identified for $\nu _h \in (2.6,3.7)$ by observing both the anomalous Hall effect and a remnant RMCD signal [35,56,73]. The ferromagnetic metal at $\nu _h=3$ can be converted to a Chern insulator with an integer quantized Hall effect by applying an out-of-plane magnetic field [35,56]. Interestingly, for a given direction of the magnetic field, the Chern number of the incipient Chern insulator at $\nu _h = 3$ has the opposite sign to that of the IQAH insulator at $\nu _h = 1$ for $\theta \ge 3.1^{\circ }$, but shares the same sign at $\theta = 2.6^{\circ }$. This observation could support the twist-angle–dependent Chern numbers of the second band obtained from large-scale DFT calculations in [50]. However, the applicability of this picture may be limited by interaction effects, which could alter the Chern number of the second band at $\nu _h=3$ compared to the DFT results obtained at $\nu _h=0$ [35]. This issue requires particular attention, as experiments in [35] reported IQSH insulators at both $\nu _h=2$ and 4 for $\theta$ between $3.0^\circ$ and $3.7^\circ$, which would imply that the first two bands in the same valley share Chern numbers of the same sign. Future work should carefully examine DFT calculations [49,50,52,54] and interaction effects to fully capture these experimental observations.

A transport study of *t*MoTe$_2$ with $\theta =2.1^{\circ }$ revealed an IQAH insulator at $\nu _h=1$, and IQSH insulators at $\nu _h=2, 4$ and 6 with two-terminal conductances of $2e^2/h$, $4e^2/h$ and $6e^2/h$, respectively [34]. The observation of these IQSH insulators is consistent with large-scale DFT calculations [50], which show that the first three moiré valence bands in $+K$ ($-K$) valleys all carry the same Chern number of $+1$ ($-1$) for $\theta$ around $\theta =2.1^{\circ }$. The multiple pairs of edge states at $\nu _h=4$ and 6 are protected by the Ising $S_z$ symmetry. Moreover, observations suggestive of a fractional quantum spin Hall (FQSH) insulator have been obtained at the odd filling factor $\nu _h = 3$, where the two-terminal conductance reaches $3e^2/h$, with each edge contributing a fractional conductance of $3e^2/2h$. This state has been shown to be incompressible with pronounced nonlocal resistance properties [34]. A proposed theoretical scenario attributes these observations to an FQSH effect at half-filling of the second moiré valence bands in both valleys in which Coulomb interactions induce correlated insulating states with fractionalized helical edge modes [34]. It should be noted that time-reversal-symmetry breaking has been observed in this state through a weak anomalous Hall effect [74], which is destroyed by an extremely tiny magnetic field of ${\sim }20$ mT. Independently, Li *et al.* [75] reported spontaneous ferromagnetism at $\nu _h=3$ for $\theta$ around $2.1^{\circ }$ using RMCD measurement and scanning nanoSQUID-on-tip magnetometry. The nanoSQUID magnetometry measurements did not detect an orbital magnetization jump, which is expected at topological gaps. The spatially resolved measurement in [75] suggests that the $\nu _h=3$ correlated topological phases could be obscured by the device disorder. Considering these observations altogether, the nature of the $\nu _h=3$ state around $\theta \approx 2.1^{\circ }$ remains unsettled.

We now turn to describe quantum phases in *t*WSe$_2$, which is a close cousin of *t*MoTe$_2$ but exhibits qualitatively different physics. A pioneering transport study [15] of *t*WSe$_2$ uncovered a correlated insulating state at $\nu _h=1$ across the twist-angle range $\theta \in (4^{\circ },5.1^{\circ })$. This correlated insulator is tunable by the applied displacement field, with the largest insulating gap appearing at a finite, $\theta$-dependent field strength. The insulator does not exhibit the quantum anomalous Hall effect, and is therefore topologically trivial. In this range of twist angles, the bandwidth of the first moiré valence band in *t*WSe$_2$ is sizable, reaching approximately 100 meV at $\theta = 5.08^\circ$ according to DFT calculations. The corresponding Coulomb interaction energy scale $e^2/(\epsilon a_M)$ is about 40 meV at $\theta = 5.08^\circ$, assuming that $\epsilon =10$, which is comparable to but smaller than the bandwidth. In this regime of moderate correlations, the Coulomb interaction is insufficient to induce valley polarization via Stoner ferromagnetism, but can stabilize intervalley-coherent antiferromagnetic states that are topologically trivial correlated insulators at $\nu _h = 1$. The $\nu _h=1$ insulating states seem to appear when the Van Hove singularities in the single-particle moiré band structure, which evolve with the displacement field, are close to the Fermi energy. When these singularities pass through $\nu _h = 1$, the diverging density of states enhances correlation effects and strengthens the insulating gap. This mechanism underlies the observed field tunability of the correlated insulator. Moreover, at $\theta =5.1^{\circ }$, zero-resistance pockets were observed on doping away from $\nu _h=1$, which appeared to indicate a transition to a superconducting state [15].

**Figure 3.**
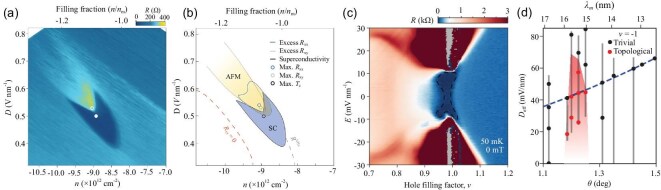
Superconductivity and topological phases observed in *t*WSe$_2$. (a) A map of the longitudinal resistance *R* is plotted as a function of the density and displacement field at $T=200\,\,\mathrm{mK}$ in *t*WSe$_2$ at $\theta =5.0^\circ$. (b) A schematic phase diagram of superconductor (SC) and antiferromagnetic (AFM) phases based on (a). Both (a) and (b) are adapted from [37]. (c) Experimental observation of superconductivity in *t*WSe$_2$ at $\theta =3.65^\circ$, reported by Xia *et al.* [38]. The plot shows the longitudinal resistance *R* as a function of the hole filling factor and applied electrical field with temperature $T=50\mathrm{mK}$. The region with zero resistance is marked with a dashed line. (d) Experimental phase diagram of the IQAH insulator at $\nu _h = 1$ as a function of $\theta$ and the effective displacement field $D_{\mathrm{eff}}$ from [70]. Red regions mark the IQAH phase, while black regions represent the topologically trivial phase. The vertical line scans across $D_{\mathrm{eff}}$ at fixed $\theta$, with dots denoting where magnetic field sweeps were used to classify the state (topological or trivial). The blue dashed line corresponds to zero tip-doping.

Robust demonstrations of superconductivity in *t*WSe$_2$ have been achieved recently. One experiment reported superconductivity in *t*WSe$_2$ at a twist angle of $5.0^{\circ }$, with a maximum critical temperature of 426 mK [37]. The superconducting phase emerges in a narrow region at finite displacement fields near $\nu _h = 1$, closely tracking the Van Hove singularities, as shown in Fig. 3a and b. Adjacent to this phase is a metallic state exhibiting Fermi surface reconstruction, likely driven by intervalley coherent antiferromagnetic order. A sharp phase boundary separates the superconducting and metallic states at low temperatures. A separate experiment reported superconductivity in *t*WSe$_2$ at smaller twist angles of $3.5^{\circ }$ and $3.65^{\circ }$ [38]. In these systems, superconductivity also emerges near $\nu _h = 1$, but around zero displacement field and away from the Van Hove singularities, as illustrated in Fig. 3c. The optimal superconducting transition temperature is about 200 mK. The superconductor borders on metallic states below and above $\nu _h=1$ at zero displacement field, but undergoes a continuous transition to a correlated insulator by increasing the displacement field. These results demonstrate a rich and tunable interplay between electronic band structure, superconductivity and correlated states in *t*WSe$_2$, highlighting phenomena that remain to be fully understood and explored.

Topological states have been observed in *t*WSe$_2$ at smaller twist angles. Local electronic compressibility measurements of *t*WSe$_2$ have been performed using scanning single-electron transistor microscopy at small $\theta$ from $1.1^{\circ }$ to $1.5^{\circ }$. [70]. Around $\theta =1.23^{\circ }$, incompressible states were identified at $\nu _h=1$ and 3, which shift in density with an applied out-of-plane magnetic field following the Streda formula in Equation (11). The persistence of these incompressible states down to zero magnetic field indicates the presence of IQAH insulators. The field dependence of their trajectories reveals that the IQAH states at $\nu _h = 1$ and 3 carry the same Chern number with a magnitude of 1. An experimental phase diagram for $\nu _h = 1$ as a function of the twist angle and tip-induced displacement field is shown in Fig. 3d. While the suppression of the IQAH phase by a finite displacement field is consistent with theoretical expectations, the emergence of the IQAH insulator within a narrow twist-angle window centered at $1.23^{\circ }$ is striking. Outside this window in Fig. 3d, Chern insulators can still be stabilized by an external magnetic field.

In a separate experiment combining magnetic-circular-dichroism and exciton-sensing techniques, Chern insulators near $\nu _h = 1$ were also observed in *t*WSe$_2$ at twist angles ranging from $1.8^{\circ }$ to $2.7^{\circ }$ under an applied magnetic field [76]. These findings are consistent with the fact that the moiré bands become progressively narrower at smaller twist angles, enhancing the tendency toward flat-band ferromagnetism and spontaneous valley polarization. This provides an explanation for the emergence of Chern insulators in *t*WSe$_2$ at small twist angles, either at zero or finite magnetic field.

IQSH effects have also been reported in *t*WSe$_2$ with $\theta =3.0^{\circ }$ at even filling factors of $\nu _h=2$ and 4 [77]. The observed signatures include (1) quantized (within 10%) resistance plateaus of $h/(\nu _h e^2)$, (2) large nonlocal transport signals, (3) an insulating bulk and (4) resistance that is insensitive to out-of-plane magnetic fields but increases under an in-plane magnetic field. These signatures are consistent with those observed in the IQSH insulators of *t*MoTe$_2$ and align with quantum transport of helical edge states in a system with spin Chern bands protected by Ising $S_z$ symmetry.

## INTEGER TOPOLOGICAL STATES

Building on the preceding overview of quantum phases in *t*MoTe$_2$ and *t*WSe$_2$, we now delve deeper into first integer and then fractional topological states, as well as superconductivity. In particular, we compare these phases to their counterparts in related systems and discuss the theoretical mechanisms underlying their emergence.

We begin with integer topological states, specifically the IQAH and IQSH insulators, which are characterized by integer-valued topological invariants. The discovery of the integer quantum Hall effect [78] in two-dimensional electron systems under strong magnetic fields marked the beginning of the study of topological electronic states in condensed matter physics. The quantization of Hall conductance in this effect is understood in terms of the Chern number $\mathcal {C}$ [79,80], which determines the Hall conductivity in units of $e^2/h$ and corresponds to the net chirality of the edge modes. Quantized Hall transport in finite-size devices can normally be understood in terms of currents carried by chiral edge states, while the bulk state is insulating. The chiral edge states are protected from backscattering and this property underlies the remarkable robustness of the quantum Hall effect.

The IQAH insulator is a generalization of the integer quantum Hall insulator [11,81–83], and is likewise characterized by a nonzero Chern number, chiral edge states and quantized Hall conductance. However, the IQAH insulator appears in the absence of an external magnetic field and therefore requires spontaneous breaking of time-reversal symmetry. IQAH insulators have been realized in magnetically doped topological insulator thin films [20], few-layer MnBi$_2$Te$_4$ [21–26], graphene-based moiré systems [27–31] and TMD-based moiré homobilayers and heterobilayers—specifically MoTe$_2$/WSe$_2$ [32].

In twisted TMD homobilayers, the valley-polarized IQAH insulator competes with other broken-symmetry states at $\nu _h=1$. This is most evident in *t*WSe$_2$, where the correlated insulators observed at $\nu _h=1$ for $\theta$ above about $3^{\circ }$ [15,37,38] are topologically trivial, although the underlying single-particle moiré bands carry valley-contrasting Chern numbers [52]. This correlated insulator is normally interpreted as a valley-coherent state with a finite ordering wave vector that is expected to result in $120^{\circ }$ antiferromagnetism in real space [9,53].

**Figure 4.**
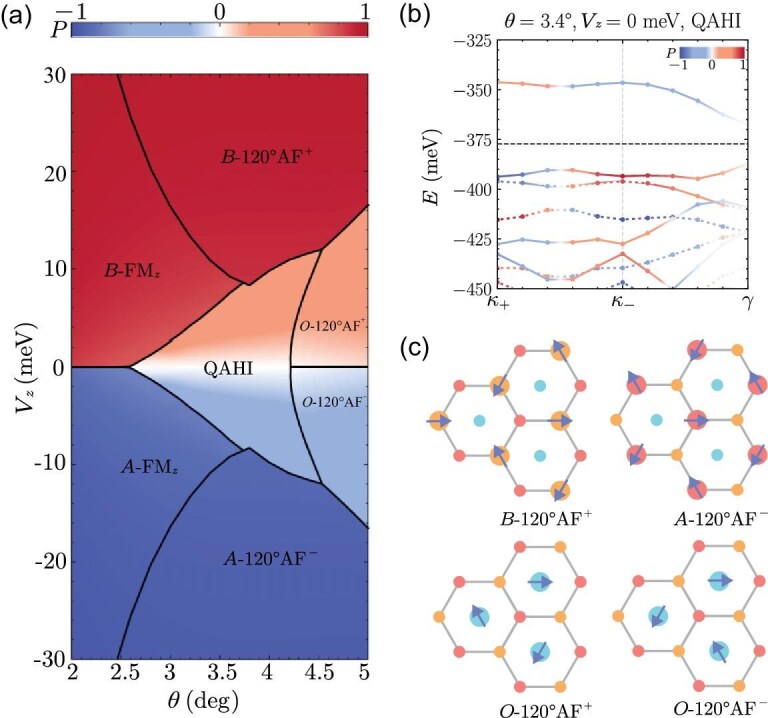
Competing topological and magnetic phases at $\nu _h = 1$. (a) Quantum phase diagram at $\nu _h = 1$ as a function of the displacement potential $V_z$ and twist angle $\theta$. The color map represents the layer polarization *P*. (b) The $\nu _h = 1$ mean-field band structure at $\theta = 3.4^\circ$ and $V_z=0\ \mathrm{meV}$ for the integer quantum anomalous Hall insulator (QAHI). The solid (dashed) lines plot bands in the $+K$ ($-K$) valley, and the horizontal black dashed line indicates the chemical potential centered in the interaction-induced gap. The color denotes the layer polarization of Bloch states, which varies dramatically across the moiré Brillouin zone at energies above the chemical potential, indicating the winding of the layer pseudospin. (c) Schematic illustration of the four $120^\circ$ antiferromagnetic states in (a) with different spatial occupations and spin vector chiralities. The red, orange and cyan dots represent the $\mathcal {R}_M^X$, $\mathcal {R}_X^M$ and $\mathcal {R}_M^M$ sites in the moiré superlattices. Here $\mathrm{AF}^{\pm }$ denote antiferromagnetic states with $\pm$ spin vector chirality. This figure is adapted from [57].

Figure 4a presents a theoretical phase diagram of *t*MoTe$_2$ at $\nu _h = 1$, obtained within the mean-field Hartree–Fock approximation [57], as a function of the twist angle $\theta$ and the interlayer potential difference $V_z$ induced by an out-of-plane displacement field. At $V_z = 0$, the phase diagram includes three distinct phases: an IQAH insulator at intermediate $\theta$ (with its mean-field band structure shown in Fig. 4b), an intervalley-coherent antiferromagnetic state at large $\theta$ and a layer-polarized ferromagnetic state at small $\theta$. At large $\theta$, weaker correlation effects favor the formation of valley-coherent states over valley-polarized ones. In contrast, at very small $\theta$, strong Coulomb interactions drive charge localization onto either $\mathcal {R}_M^X$ or $\mathcal {R}_X^M$ sites, leading to spontaneous layer polarization. The phase boundaries are also sensitive to $V_z$: when $V_z$ exceeds critical values, the IQAH phase is replaced by a layer-polarized, topologically trivial correlated insulator similar to those that appear in extended Hubbard models on triangular lattices, which can be independently realized in moiré TMD heterobilayers [84–86]. We note that this mean-field phase diagram is qualitative, since it neglects effects like lattice relaxation and beyond-mean-field correlations. Experimental signatures of the competition between the valley polarized states and other magnetic states in *t*MoTe$_2$ have been reported in [87]. Because the topological and magnetic phases shown in Fig. 4 are symmetry-breaking phases driven by interactions, they host a variety of low-energy collective excitations, such as excitons, magnons and domain walls, which are currently under active investigation [88–92].

The IQSH insulator is a generalization of the IQAH insulator [12,93,94], characterized by topological bands with spin-contrasting Chern numbers. The IQSH state is bulk insulating and, when protected by Ising spin symmetry, can host multiple helical edge channels. If Ising spin symmetry is broken but time-reversal symmetry is preserved, the IQSH can become a time-reversal invariant two-dimensional topological insulator, classified by a $Z_2$ topological invariant [95]. In this case, time-reversal symmetry protects a single helical mode per edge, consisting of a pair of counterpropagating edge states with opposite spins. For symmetry-preserving disorder, the helical modes are also immune to backscattering. Therefore, each helical mode contributes $e^2/h$ to the two-terminal conductance. Similar transport signatures of two-dimensional topological insulators have been previously reported in HgTe quantum wells [96], InAs/GaSb quantum wells [97,98] and monolayer WTe$_2$ [99,100].

In twisted TMD homobilayers, the total spin $S_z$ is approximately conserved and can serve as an emergent effective symmetry. The IQSH insulator at $\nu _h = 2$ exhibits a two-terminal conductance of $2e^2/h$, protected by both $S_z$ and time-reversal symmetry. In contrast, the IQSH insulators at $\nu _h = 4$ and 6 exhibit two-terminal conductance values of $\nu _h e^2/h$, arising from multiple helical edge modes protected solely by the approximate $S_z$ symmetry. We also note that other competing states may exist at $\nu _h = 2$ [53,101,102], but experimental evidence for these is so far lacking.

## FRACTIONALIZED TOPOLOGICAL STATES

Two-dimensional electron systems under strong magnetic fields exhibit not only the integer quantum Hall effect but also the fractional quantum Hall effect [103], a paradigmatic example of a strongly correlated topological phase. At certain fractional Landau-level fillings, electron interactions stabilize incompressible quantum liquids with fractionally quantized Hall conductance. A prototypical example is the fractional quantum Hall insulator (FQHI) at filling factor $1/3$, which exhibits a Hall conductivity of $(1/3)e^2/h$ and is well described by the Laughlin wave function [104]. FQHIs occur at various rational fillings with odd denominators—such as $1/3$, $2/5$ and $3/7$—and less commonly at even-denominator fractions like $5/2$. These phases host quasiparticle excitations with fractional charge and anyonic statistics, as exemplified by the Laughlin quasiparticles and quasiholes in the $1/3$ state.

The concept of FQHIs was first extended to the zero-magnetic-field case in a series of theoretical studies of model lattice systems [105–109]. The zero-field states were referred to as fractional Chern insulators (FCIs). These studies focused on systems with partially filled topological flat bands with nonzero Chern numbers that emulate Landau levels, allowing strong electron interactions to stabilize fractionalized phases analogous to those in the FQHI. FCIs exhibit key hallmarks of fractional quantum Hall physics—including fractionally quantized Hall conductance, anyonic excitations and topological ground-state degeneracy—but can also display new features, going beyond the physics of Landau levels [110].

The definition of FCIs was later expanded to include lattice-like experimental systems at finite magnetic fields. FCIs have been observed in several van der Waals heterostructures. Magnetocapacitance measurements have provided evidence of FCIs at fractional fillings of Harper–Hofstadter bands, arising from the interplay of a strong magnetic field ($\sim$30 T) and a superlattice potential in a bilayer graphene–hexagonal boron nitride heterostructure [111]. In magic-angle twisted bilayer graphene, FCIs have been reported at lower magnetic fields ($\sim$5 T) through high-resolution local compressibility measurements [112]. FCIs at zero magnetic field, known as FQAH insulators, were first realized in *t*MoTe$_2$ [16–19], a phenomenon now well established through various experimental techniques. FQAH insulators have also been observed in another moiré system, multilayer rhombohedral graphene aligned with hexagonal boron nitride [113,114].

**Figure 5.**
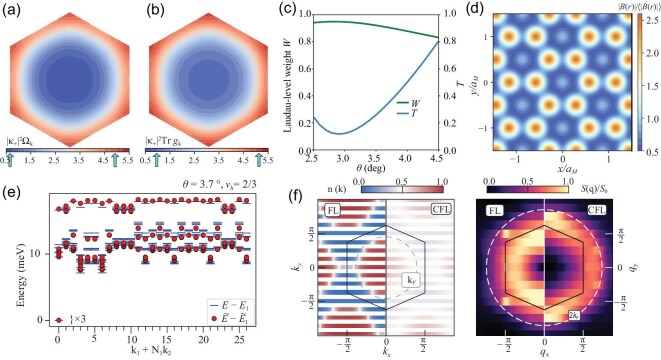
Numerical results in the first moiré valence band of *t*MoTe$_2$ for fractionalized states. (a and b) Berry curvature $\Omega _{{k}}$ and the trace of the quantum metric $\mathrm{Tr} g_{{k}}$ at $\theta = 2.9^\circ$ in the moiré Brillouin zone. (c) Deviation *T* from ideal quantum geometry and Laudau-level weight *W* as functions of $\theta$. (d) Map of $|\mathcal {B}({r})|$ scaled by its spatial average at $\theta = 2.9^\circ$. (e) ED spectra based on $\varphi _{1,{k}}$ (blue lines) and $\Theta _{{k}}({r})$ (red dots) at $\nu _h = 2/3$ and $\theta = 3.7^\circ$. (f) Infinite DMRG results of the composite Fermi liquid (CFL) at $\nu _h = 1/2$ and $\theta = 3.7^\circ$. The left panel shows the occupations $n({k})$ in the Brillouin zone, comparing the Fermi liquid (left side) and the CFL (right side). The right panel presents the connected structure factor $S({q}) = \langle \hat{\rho }_{{q}} \hat{\rho }_{-{q}} \rangle - \langle \hat{\rho }_{{q}} \rangle \langle \hat{\rho }_{-{q}} \rangle$. Panels (a–e) are adapted from [45], and panel (f) is from [68].

The emergence of FQAH insulators within a valley-polarized Chern band in *t*MoTe$_2$ can be theoretically understood by mapping the band to Landau levels, a process that can be achieved through two complementary approaches. One approach utilizes the adiabatic approximation, which incorporates the emergent magnetic field $B_{\mathrm{eff}}$ generated by the skyrmion texture, shown in Equation (8), in the moiré Hamiltonian [44,45,115]. A related approach is based on the observation that the first moiré band in *t*MoTe$_2$ has nearly ideal quantum geometry, in which the Berry curvature $\Omega _{\boldsymbol k}$ and the trace of the quantum metric $\mathrm{Tr} g_{\boldsymbol k}$ fluctuate in sync in momentum space, as illustrated in Fig. 5a and b. A measure of the deviation from ideal quantum geometry is $T=({1}/{2\pi })\int d^2\boldsymbol{k}\mathrm{Tr}\, g_{\boldsymbol{k}}-|\mathcal {C}|$, where momentum integration is over the moiré Brillouin zone, $\mathcal {C}$ is the Chern number and *T* can be shown to be non-negative. A band with ideal quantum geometry has $T=0$ and can be shown to have generalized Landau-level Bloch wave functions [116,117] of the form


(14) \begin{eqnarray*}
\Theta _{\boldsymbol{k}}(\boldsymbol{r})=\mathcal {N}_{\boldsymbol{k}}\mathcal {B}(\boldsymbol{r})\Psi _{0,\boldsymbol{k}}(\boldsymbol{r}),
\end{eqnarray*}

where $\Psi _{0,\boldsymbol{k}} (\boldsymbol{r})$ is the magnetic Bloch wave function of the zeroth Landau level, $\mathcal {B}(\boldsymbol{r})$ is spatially dependent but wave-vector $\boldsymbol k$-independent and $\mathcal {N}_{\boldsymbol{k}}$ is a normalization factor. In *t*MoTe$_2$, *T* is not zero but can be as small as 0.1, as shown in Fig. 5c. The wave function $\varphi _{1,\boldsymbol{k}}$ of the first moiré valence band in *t*MoTe$_2$ can be approximated by $\Theta _{\boldsymbol{k}}$. A variational approach, developed in [45], is used to obtain the optimal $\mathcal {B}(\boldsymbol{r})$ by maximizing the Landau-level weight *W*, defined as the momentum average of $|\langle \psi _{\boldsymbol {k}} | \Theta _{\boldsymbol{k}} \rangle |^2$. The maximized weight *W* is shown in Fig. 5c, which reaches up to about 0.95. The obtained $\mathcal {B}(\boldsymbol{r})$ function is plotted in Fig. 5d and has a spatial variation that matches that of the total electron density of the first moiré band.

The ideal Chern band with the wave function $\Theta _{\boldsymbol{k}}(\boldsymbol{r})$ possesses a distinctive feature: it enables the construction of trial wave functions for FCIs [116,117]. These trial states take the form $\Phi _F=\Psi _F\prod _{i} \mathcal {B}(\boldsymbol{r}_i)$, where $\boldsymbol{r}_i$ denotes the coordinate of the *i*th electron, and $\Psi _F$ describes fractional quantum Hall states (or composite Fermi liquid states) within the zeroth Landau level. These constructed wave functions $\Phi _F$ are exact ground states for specific short-range repulsive Hamiltonians [117]. The substantial overlap between the single-particle states $\varphi _{1,\boldsymbol{k}}$ and the generalized Landau-level functions $\Theta _{\boldsymbol{k}}(\boldsymbol{r})$ offers a simple physical picture that explains the emergence of FQAH insulators in *t*MoTe$_2$. Figure 5e presents the exact diagonalization (ED) spectra at $\nu _h=2/3$ respectively based on $\varphi _{1,\boldsymbol{k}}$ and $\Theta _{\boldsymbol{k}}(\boldsymbol{r})$ using Coulomb interaction, which agree quantitatively and clearly reveal an FCI state.

Numerical studies are essential for investigating FCIs. While ED studies are limited by small system sizes and few-band projections, they have proven successful in identifying FCIs through analysis of the energy spectra and quasi-degeneracy of ground states, spectral flow under flux insertion and the unique counting of the entanglement spectrum below the gap [13,45–48,51,68,69,118–121]. The density matrix renormalization group (DMRG) method has also been applied to study fractionalized states in *t*MoTe$_2$ [68,122,123]. For example, numerical evidence of the composite Fermi liquid at $\nu _h=1/2$ in *t*MoTe$_2$ from DMRG calculations is shown in Fig. 5f [68]. Recently, a deep learning quantum Monte Carlo method based on neural networks has been developed to study both the integer and fractional topological states in *t*MoTe$_2$ [124,125].

The presence of spin-contrasting Chern bands in *t*MoTe$_2$ yields physics that goes beyond the Landau-level framework, with the experimental signature of the FQSH insulator at $\nu _h = 3$ serving as a prominent example [34]. This has prompted several theoretical studies to explore the nature of this state [126–131], with candidate states such as non-Abelian spin Hall insulators and Halperin states. DFT band-structure calculations [50,52,54,132] have shown that the second moiré band in *t*MoTe$_2$ at $\theta$ around $2^{\circ }$ can mimic the first Landau level. Numerical evidence of non-Abelian fractionalized states at half-filling of the second moiré band, with spontaneous valley polarization, has been reported in *t*MoTe$_2$ [54,132–135]. These findings suggest the possibility, not yet realized, that *t*MoTe$_2$ might be a platform for exploring non-Abelian topological phases.

The low-energy collective modes of insulators can play a helpful role in characterizing phases of matter, especially at long wavelengths where they couple directly to light. Numerical calculations have established [136–138] that the FCI states of *t*MoTe$_2$ have low-energy intraband collective excitations that are similar in many respects to the magnetoroton excitations of conventional fractional quantum Hall systems. In the FQHI case, magnetoroton excitations [139] are optically dark, i.e. they do not contribute to the optical conductivity. In the FCI case, it is easy to see [140] that they are dark in the case of perfectly flat bands since the projection of the current operator to the partially occupied band is zero. It has been argued that the coupling to photons is anomalously weak [140] even when the bands are not perfectly flat, but it can be strengthened [141,142] by adding periodic modulation at length scales that are longer than the moiré period. Even if optical signals are weak, they might still be observable and highly informative. Collective-mode studies of FCIs therefore have considerable promise, but require progress in making larger devices, improving the energy resolution of near-field optical probes and/or advancing techniques for terahertz spectroscopy studies of small devices [143].

## SUPERCONDUCTORS

The observation of superconductivity in *t*WSe$_2$ across multiple devices and twist angles establishes twisted homobilayer TMDs as a second moiré platform, after twisted graphene bilayers and multilayers, that supports robust superconductivity [37,38]. Available experiments show that *t*WSe$_2$ can host superconductivity at $\theta =3.5^{\circ }$, $3.65^{\circ }$ and $5^{\circ }$, suggesting that superconductivity in this system is less sensitive to the twist angle compared to graphene-based moiré systems. This behavior reflects a general trend in twisted TMD homobilayers, where many-body correlation effects persist over a wider range of twist angles due to a weaker dependence of the moiré bandwidth on $\theta$. Because correlations are weak away from the narrow *magic* twist-angle regime, it is more difficult to precisely reproduce behavior from one device to another in the graphene multilayer case. Nevertheless, it is important to recognize that significant differences exist between *t*WSe$_2$ near $\theta = 5^{\circ }$ and near $\theta = 3.5^{\circ }$, indicating that the nature of the superconducting state may vary across this range. At $\theta \approx 5^{\circ }$, superconductivity in *t*WSe$_2$ emerges in a narrow region at finite displacement fields, closely following Van Hove singularities, and is adjacent to a metallic phase exhibiting Fermi surface reconstruction driven by intervalley coherent antiferromagnetic order [37]. In contrast, at twist angles near $3.5^{\circ }$, superconductivity appears around zero displacement field and $\nu _h=1$, away from Van Hove singularities, and is adjacent to a correlated insulator at a finite displacement field [38]. These differences suggest that superconductivity in *t*WSe$_2$ near $\theta = 5^{\circ }$ is likely driven by a weak-coupling pairing mechanism arising from Fermi surface instability, whereas that near $\theta = 3.5^{\circ }$ is more likely governed by a strong-coupling mechanism. A common finding in the two experiments of Guo *et al.* [37] and Xia *et al.* [38] is that superconductivity can emerge from a symmetry-unbroken normal state, suggesting pairing between the two opposite valleys that are time-reversal partners—i.e. intervalley pairing. A variety of theoretical approaches have been employed to explore the microscopic origin of superconductivity in the different twist-angle regimes [144–151]. If these two limits are connected continuously in the parameter space of $\theta$, $\nu _h$ and the displacement field, *t*WSe$_2$ could act as an attractive laboratory to study the crossover between superconductivity at weak and strong coupling.

The superconductivity reported in *t*MoTe$_2$ at $\theta =3.83^{\circ }$ has notably different characteristics [36]. It is embedded in a ferromagnetic metal and adjacent to the FQAH insulator at $\nu _h=2/3$ and the reentrant IQAH insulator. Above the superconducting transition temperature, *t*MoTe$_2$ enters a metallic phase with an anomalous Hall effect and magnetic hysteresis, indicating the emergence of superconductivity from a valley-polarized ferromagnetic parent state. This suggests that the electron pairing is within a spin-/valley-polarized band. Similar superconductivity, emerging out of a ferromagnetic metal with an anomalous Hall effect and magnetic hysteresis, has also been observed in moiréless rhombohedral multilayer graphene, although not in proximity to an FQAH or IQAH insulator [152].

The theoretical implications of pairing within a valley-polarized, ferromagnetic state are profound. In conventional superconductors such as aluminum, time-reversal symmetry plays a central role in stabilizing spin-singlet Cooper pairing of time-reversed states. In *t*MoTe$_2$ time-reversal symmetry is already broken in the normal state, and pairing must occur within a single spin-polarized band, so that the same-spin pairing channel is the only one available. This superconductor is believed to be chiral because it should have finite orbital magnetization for pairing within the same valley [152,153]. If the superconducting state is gapped, as suggested in [154], the Bogoliubov–de Gennes Hamiltonian can host bulk bands with finite Chern numbers and gapless Majorana edge modes. The close proximity between the superconducting phase and the FQAH insulator in *t*MoTe$_2$ further raises questions about their potential microscopic connection. In this context, the theoretical possibility of anyon superconductivity—a phase in which fractionalized quasiparticle excitations of a parent FQAH state condense into a superconducting order—has attracted growing interest [155–159].

## OUTLOOK

Twisted TMD homobilayers represent a new frontier in the study of correlated and topological phases in two-dimensional systems. The combination of strong spin-orbit coupling, moiré flat bands with valley-contrasting topological properties and pronounced electron-electron interactions has enabled the discovery of a remarkably rich variety of emergent quantum phases. Going forward, there are many open directions for both experiment and theory that will not only deepen our understanding of the underlying physics but may also lead to opportunities for realizing new quantum phases of matter, and for materials and quantum device engineering.

On the experimental front, improving device quality may uncover new fragile and exotic quantum phases. A persistent challenge in twistronics is the inevitability of atomic-scale disorder, twist-angle inhomogeneity and nonuniform strain—all of which can suppress energy gaps, obscure quantization and hide symmetry-breaking phenomena. In particular, limited mobility often leads to a finite longitudinal resistance that masks the dissipationless nature expected in ideal quantum Hall states. Insights from fractional quantum Hall systems suggest that many delicate phases emerge only when mobility exceeds a critical threshold [160]. Achieving high mobility and low contact resistance in twisted TMD homobilayers is similarly crucial. Two independent transport measurements of *t*MoTe$_2$ with improved sample quality have achieved a dissipationless FQAH effect with vanishing longitudinal resistance [161], and have separately revealed superconductivity adjacent to the FQAH insulator [36], underscoring the key role of device quality.

A key direction for future research is the systematic exploration and classification of the correlated and topological phases that emerge in twisted TMDs as functions of the twist angle, carrier density, electric displacement field, magnetic field and applied pressure. Thus far, only a subset of twist angles have been investigated in detail. Systematic studies may reveal new quantum states, competing orders or phase transitions. For example, non-Abelian fractionalized states with spontaneous valley polarization have been numerically predicted in *t*MoTe$_2$ at half-filling of the second moiré valence bands near $\theta \approx 2.0^{\circ }$ [54,132–135], but await experimental confirmation. Similarly, pressure has been predicted to stabilize the FQAH insulator in *t*WSe$_2$ [119], motivating further experimental investigation in this direction.

A major open question is the direct detection of charge fractionalization and anyonic statistics of quasiparticles in the FQAH insulator in *t*MoTe$_2$. Probing fractional charge via shot-noise [162,163] or scanning-tunneling measurements [164], and detecting fractional statistics through interferometry [165] or anyon-collider measurements [166], are essential next steps. Realizing such measurements in *t*MoTe$_2$ would further establish the FQAH state as a fractional topological phase and mark an important step in accessing and manipulating anyonic excitations without an external magnetic field. Moreover, if non-Abelian fractionalized states could be realized in *t*MoTe$_2$, these techniques would be indispensable for advancing toward fault-tolerant quantum computation [167].

Superconductivity in twisted TMD homobilayers is another fertile area for future exploration. It has been observed in both *t*WSe$_2$ and *t*MoTe$_2$, appearing in proximity to correlated states or even fractional topological phases [36–38]. However, the nature of the superconducting state and the pairing mechanism remains unresolved. Experimental techniques such as tunneling spectroscopy, Josephson junction measurements, magneto-optical spectroscopy and thermal conductivity could shed light on the pairing symmetry. Applying in-plane magnetic fields may tune the superconducting state [168,169], while angle-resolved measurements of in-plane critical currents can probe nematicity and the superconducting diode effect. Furthermore, junctions formed between superconducting regions and integer or fractional topological states—such as IQAH, IQSH or FQAH insulators—provide a promising platform for engineering exotic quasiparticles, including Majorana zero modes [101] and parafermions [170].

Theoretical progress will be essential to guide and interpret the growing body of experimental discoveries in twisted TMD homobilayers. Continuum models have been widely used to reveal key qualitative features, such as flat moiré bands and the emergence of topological Chern bands. On the other hand, DFT calculations are indispensable in quantifying the role of lattice relaxation in moiré superlattices and its influence on the twist-angle dependence of the electronic structure. However, DFT studies of large moiré unit cells are computationally demanding and often require approximations or downfolding strategies to extract effective models. Nevertheless, they are crucial for validating and refining continuum models. Systematic refinement of these models—alongside *ab initio*–informed parameterization—remains essential to bridge theory with experiment and identify realistic conditions under which interaction-driven phenomena can emerge.

Beyond single-particle descriptions, uncovering correlated and topological phases in moiré TMDs requires a diverse set of many-body methods, including mean-field theories, exact diagonalization, DMRG and quantum Monte Carlo. Each approach provides valuable, often complementary insights. A coherent understanding of IQAH and FQAH insulators is gradually emerging through these techniques. However, developing a controlled, microscopic theory of superconductivity in these systems remains a critical challenge. Addressing this challenge will require systematic benchmarking of numerical results against each other and experimental data. Additionally, careful analysis of simplified yet realistic models will be essential to isolate and identify the key physical mechanisms at play. The unique ability to tune and follow the evolution of electronic properties as the twist angle and carrier density are changed could make it easier to identify trends and establish rules of thumb that apply outside of the moiré-materials world.

Moiré materials offer exciting opportunities to observe topological phenomena beyond the Landau-level framework. Graphene and TMD-based moiré materials host valley-contrasting Chern bands, paving the way for the realization of novel topological states such as FQSH insulators, with evidence reported in *t*MoTe$_2$ [34]. Furthermore, the topological bands in these systems exhibit nonuniform quantum geometric tensors, finite bandwidths and varying Chern numbers—features not present in Landau levels. These characteristics enable the exploration of new phenomena beyond Landau-level physics, with the recently observed superconductivity in *t*MoTe$_2$ [36] serving as an important example. Such developments highlight the potential for discovering novel quantum phases that extend the boundaries of traditional topological theories established in Landau levels.

In the long term, twisted TMD homobilayers could give rise to a new class of functional quantum materials, leveraging the unique capability of a single device to host a wide range of electrically tunable quantum phases. The realization of topologically protected edge states and domain wall modes—manipulable by electric and magnetic fields—can offer a path toward gate-defined topological circuits with low dissipation. Simultaneously, the emergence of gate-tunable superconductivity enables the design of moiré-based Josephson junctions and hybrid platforms that combine superconducting and topological states, potentially supporting non-Abelian excitations for topological quantum computation. Beyond the materials themselves, the novel quantum phenomena observed in twisted TMDs can inspire the design and engineering of quantum phases across a range of platforms, including other moiré systems, atomic scale solid-state materials, cold atoms in optical lattices and quantum computing platforms such as superconducting circuits or Rydberg atoms.

## References

[1] Bistritzer R, MacDonald AH. Moiré bands in twisted double-layer graphene. Proc Natl Acad Sci USA 2011; 108: 12233–7. 10.1073/pnas.1108174108 21730173 PMC3145708

[2] Cao Y, Fatemi V, Demir A et al. Correlated insulator behaviour at half-filling in magic-angle graphene superlattices. Nature 2018; 556: 80–4. 10.1038/nature26154 29512654

[3] Cao Y, Fatemi V, Fang S et al. Unconventional superconductivity in magic-angle graphene superlattices. Nature 2018; 556: 43–50. 10.1038/nature26160 29512651

[4] Andrei EY, MacDonald AH. Graphene bilayers with a twist. Nat Mater 2020; 19: 1265–75. 10.1038/s41563-020-00840-0 33208935

[5] Balents L, Dean CR, Efetov DK et al. Superconductivity and strong correlations in moiré flat bands. Nat Phys 2020; 16: 725–33. 10.1038/s41567-020-0906-9

[6] Mak KF, Shan J. Semiconductor moiré materials. Nat Nanotechnol 2022; 17: 686–95. 10.1038/s41565-022-01165-6 35836003

[7] Nuckolls KP, Yazdani A. A microscopic perspective on moiré materials. Nat Rev Mater 2024; 9: 460–80. 10.1038/s41578-024-00682-1

[8] Xiao D, Liu GB, Feng W et al. Coupled spin and valley physics in monolayers of MoS$_2$ and other group-VI dichalcogenides. Phys Rev Lett 2012; 108: 196802. 10.1103/PhysRevLett.108.196802 23003071

[9] Pan H, Wu F, Das Sarma S. Band topology, Hubbard model, Heisenberg model, and Dzyaloshinskii-Moriya interaction in twisted bilayer WSe_2_. Phys Rev Res 2020; 2: 033087. 10.1103/PhysRevResearch.2.033087

[10] Wu F, Lovorn T, Tutuc E et al. Topological insulators in twisted transition metal dichalcogenide homobilayers. Phys Rev Lett 2019; 122: 086402. 10.1103/PhysRevLett.122.086402 30932597

[11] Haldane FDM. Model for a quantum Hall effect without Landau levels: condensed-matter realization of the ‘parity anomaly’. Phys Rev Lett 1988; 61: 2015–8. 10.1103/PhysRevLett.61.2015 10038961

[12] Kane CL, Mele EJ. Quantum spin Hall effect in graphene. Phys Rev Lett 2005; 95: 226801. 10.1103/PhysRevLett.95.226801 16384250

[13] Li H, Kumar U, Sun K et al. Spontaneous fractional Chern insulators in transition metal dichalcogenide moiré superlattices. Phys Rev Res 2021; 3: L032070. 10.1103/PhysRevResearch.3.L032070

[14] Devakul T, Crépel V, Zhang Y et al. Magic in twisted transition metal dichalcogenide bilayers. Nat Commun 2021; 12: 6730. 10.1038/s41467-021-27042-9 34795273 PMC8602625

[15] Wang L, Shih EM, Ghiotto A et al. Correlated electronic phases in twisted bilayer transition metal dichalcogenides. Nat Mater 2020; 19: 861–6. 10.1038/s41563-020-0708-6 32572205

[16] Cai J, Anderson E, Wang C et al. Signatures of fractional quantum anomalous Hall states in twisted MoTe_2_. Nature 2023; 622: 63–8. 10.1038/s41586-023-06289-w 37315640

[17] Zeng Y, Xia Z, Kang K et al. Thermodynamic evidence of fractional Chern insulator in moiré MoTe_2_. Nature 2023; 622: 69–73. 10.1038/s41586-023-06452-3 37494955

[18] Park H, Cai J, Anderson E et al. Observation of fractionally quantized anomalous Hall effect. Nature 2023; 622: 74–9. 10.1038/s41586-023-06536-0 37591304

[19] Xu F, Sun Z, Jia T et al. Observation of integer and fractional quantum anomalous Hall effects in twisted bilayer MoTe_2_. Phys Rev X 2023; 13: 031037.

[20] Chang CZ, Zhang J, Feng X et al. Experimental observation of the quantum anomalous Hall effect in a magnetic topological insulator. Science 2013; 340: 167–70. 10.1126/science.1234414 23493424

[21] Zhao YF, Zhang R, Mei R et al. Tuning the Chern number in quantum anomalous Hall insulators. Nature 2020; 588: 419–23. 10.1038/s41586-020-3020-3 33328665

[22] Deng Y, Yu Y, Shi MZ et al. Quantum anomalous Hall effect in intrinsic magnetic topological insulator MnBi_2_Te_4_. Science 2020; 367: 895–900. 10.1126/science.aax8156 31974160

[23] Liu C, Wang Y, Li H et al. Robust axion insulator and Chern insulator phases in a two-dimensional antiferromagnetic topological insulator. Nat Mater 2020; 19: 522–7. 10.1038/s41563-019-0573-3 31907415

[24] Zhang D, Shi M, Zhu T et al. Topological axion states in the magnetic insulator MnBi_2_Te_4_ with the quantized magnetoelectric effect. Phys Rev Lett 2019; 122: 206401. 10.1103/PhysRevLett.122.206401 31172761

[25] Li J, Li Y, Du S et al. Intrinsic magnetic topological insulators in van der Waals layered MnBi_2_Te_4_-family materials. Sci Adv 2019; 5: eaaw5685. 10.1126/sciadv.aaw5685 31214654 PMC6570506

[26] Otrokov MM, Klimovskikh II, Bentmann H et al. Prediction and observation of an antiferromagnetic topological insulator. Nature 2019; 576: 416–22. 10.1038/s41586-019-1840-9 31853084

[27] Sharpe AL, Fox EJ, Barnard AW et al. Emergent ferromagnetism near three-quarters filling in twisted bilayer graphene. Science 2019; 365: 605–8. 10.1126/science.aaw3780 31346139

[28] Serlin M, Tschirhart CL, Polshyn H et al. Intrinsic quantized anomalous Hall effect in a moiré heterostructure. Science 2020; 367: 900–3. 10.1126/science.aay5533 31857492

[29] Chen G, Sharpe AL, Fox EJ et al. Tunable correlated Chern insulator and ferromagnetism in a moiré superlattice. Nature 2020; 579: 56–61. 10.1038/s41586-020-2049-7 32132694

[30] Polshyn H, Zhu J, Kumar MA et al. Electrical switching of magnetic order in an orbital Chern insulator. Nature 2020; 588: 66–70. 10.1038/s41586-020-2963-8 33230333

[31] Polshyn H, Zhang Y, Kumar MA et al. Topological charge density waves at half-integer filling of a moiré superlattice. Nat Phys 2022; 18: 42–7. 10.1038/s41567-021-01418-6

[32] Li T, Jiang S, Shen B et al. Quantum anomalous Hall effect from intertwined moiré bands. Nature 2021; 600: 641–6. 10.1038/s41586-021-04171-1 34937897

[33] Anderson E, Cai J, Reddy AP et al. Trion sensing of a zero-field composite Fermi liquid. Nature 2024; 635: 590–5. 10.1038/s41586-024-08134-0 39567789

[34] Kang K, Shen B, Qiu Y et al. Evidence of the fractional quantum spin Hall effect in moiré MoTe_2_. Nature 2024; 628: 522–6. 10.1038/s41586-024-07214-5 38509375

[35] Xu F, Chang X, Xiao J et al. Interplay between topology and correlations in the second moiré band of twisted bilayer MoTe$_2$. Nat Phys 2025; 21: 542–8. 10.1038/s41567-025-02803-1

[36] Fan X, Sun Z, Li J et al. Signatures of unconventional superconductivity near reentrant and fractional quantum anomalous Hall insulators [preprint]. arXiv: 2025.06972.

[37] Guo Y, Pack J, Swann J et al. Superconductivity in 5.0$^\circ$ twisted bilayer WSe_2_. Nature 2025; 637: 839–45. 10.1038/s41586-024-08381-1 39843588

[38] Xia Y, Han Z, Watanabe K et al. Superconductivity in twisted bilayer WSe_2_. Nature 2025; 637: 833–8. 10.1038/s41586-024-08116-2 39478226

[39] Zhang YH, Sheng DN, Vishwanath A. SU(4) chiral spin liquid, exciton supersolid, and electric detection in moiré bilayers. Phys Rev Lett 2021; 127: 247701. 10.1103/PhysRevLett.127.247701 34951785

[40] Xu Y, Kang K, Watanabe K et al. A tunable bilayer Hubbard model in twisted WSe$_2$. Nat Nanotechnol 2022; 17: 934–9. 10.1038/s41565-022-01180-7 35915334

[41] Kuhlenkamp C, Kadow W, Imamoğlu A et al. Chiral pseudospin liquids in moiré heterostructures. Phys Rev X 2024; 14: 021013.

[42] Angeli M, MacDonald AH. $\Gamma$ valley transition metal dichalcogenide moiré bands. Proc Natl Acad Sci USA 2021; 118: e2021826118. 10.1073/pnas.2021826118 33658375 PMC7958387

[43] Yu H, Chen M, Yao W. Giant magnetic field from moiré induced Berry phase in homobilayer semiconductors. Natl Sci Rev 2019; 7: 12–20. 10.1093/nsr/nwz117 35296065 PMC8559909

[44] Morales-Durán N, Wei N, Shi J et al. Magic angles and fractional Chern insulators in twisted homobilayer transition metal dichalcogenides. Phys Rev Lett 2024; 132: 096602. 10.1103/PhysRevLett.132.096602 38489616

[45] Li B, Wu F. Variational mapping of Chern bands to Landau levels: application to fractional Chern insulators in twisted MoTe_2_. Phys Rev B 2025; 111: 125122. 10.1103/PhysRevB.111.125122

[46] Reddy AP, Alsallom F, Zhang Y et al. Fractional quantum anomalous Hall states in twisted bilayer MoTe_2_ and WSe_2_. Phys Rev B 2023; 108: 085117. 10.1103/PhysRevB.108.085117

[47] Wang C, Zhang XW, Liu X et al. Fractional Chern insulator in twisted bilayer MoTe_2_. Phys Rev Lett 2024; 132: 036501. 10.1103/PhysRevLett.132.036501 38307072

[48] Xu C, Li J, Xu Y et al. Maximally localized Wannier functions, interaction models, and fractional quantum anomalous Hall effect in twisted bilayer MoTe_2_. Proc Natl Acad Sci USA 2024; 121: e2316749121. 10.1073/pnas.2316749121 38349878 PMC10895274

[49] Mao N, Xu C, Li J et al. Transfer learning relaxation, electronic structure and continuum model for twisted bilayer MoTe$_2$. Commun Phys 2024; 7: 262. 10.1038/s42005-024-01754-y

[50] Zhang XW, Wang C, Liu X et al. Polarization-driven band topology evolution in twisted MoTe_2_ and WSe_2_. Nat Commun 2024; 15: 4223. 10.1038/s41467-024-48511-x 38762554 PMC11102499

[51] Jia Y, Yu J, Liu J et al. Moiré fractional Chern insulators. I. First-principles calculations and continuum models of twisted bilayer MoTe_2_. Phys Rev B 2024; 109: 205121. 10.1103/PhysRevB.109.205121

[52] Zhang Y, Pi H, Liu J et al. Universal moiré-model-building method without fitting: application to twisted MoTe$_2$ and WSe$_2$ [preprint]. arXiv: 2024.08108.

[53] Qiu WX, Li B, Luo XJ et al. Interaction-driven topological phase diagram of twisted bilayer MoTe_2_. Phys Rev X 2023; 13: 041026.

[54] Xu C, Mao N, Zeng T et al. Multiple Chern bands in twisted MoTe_2_ and possible non-Abelian states. Phys Rev Lett 2025; 134: 066601. 10.1103/PhysRevLett.134.066601 40021170

[55] Anderson E, Fan FR, Cai J et al. Programming correlated magnetic states with gate-controlled moiré geometry. Science 2023; 381: 325–30. 10.1126/science.adg4268 37347950

[56] Park H, Cai J, Anderson E et al. Ferromagnetism and topology of the higher flat band in a fractional Chern insulator. Nat Phys 2025; 21: 549–55. 10.1038/s41567-025-02804-0

[57] Li B, Qiu WX, Wu F. Electrically tuned topology and magnetism in twisted bilayer MoTe_2_ at ν_*h*_ = 1. Phys Rev B 2024; 109: L041106. 10.1103/PhysRevB.109.L041106

[58] Středa P, Smrčka L. Galvanomagnetic effects in alloys in quantizing magnetic fields. Phys Status Solidi (B) 1975; 70: 537–48. 10.1002/pssb.2220700213

[59] Redekop E, Zhang C, Park H et al. Direct magnetic imaging of fractional Chern insulators in twisted MoTe$_2$. Nature 2024; 635: 584–9. 10.1038/s41586-024-08153-x 39567790

[60] MacDonald AH. Introduction to the physics of the quantum Hall regime [preprint]. arXiv: cond-mat/9410047.

[61] Ji Z, Park H, Barber ME et al. Local probe of bulk and edge states in a fractional Chern insulator. Nature 2024; 635: 578–83. 10.1038/s41586-024-08092-7 39567787

[62] Willett R, Eisenstein JP, Störmer HL et al. Observation of an even-denominator quantum number in the fractional quantum Hall effect. Phys Rev Lett 1987; 59: 1776–9. 10.1103/PhysRevLett.59.1776 10035326

[63] Jain JK. Composite-fermion approach for the fractional quantum Hall effect. Phys Rev Lett 1989; 63: 199–202. 10.1103/PhysRevLett.63.199 10040805

[64] Halperin BI, Lee PA, Read N. Theory of the half-filled Landau level. Phys Rev B 1993; 47: 7312–43. 10.1103/PhysRevB.47.7312 10004728

[65] Willett RL, Ruel RR, West KW et al. Experimental demonstration of a Fermi surface at one-half filling of the lowest Landau level. Phys Rev Lett 1993; 71: 3846–9. 10.1103/PhysRevLett.71.3846 10055088

[66] Kang W, Stormer HL, Pfeiffer LN et al. How real are composite fermions? Phys Rev Lett 1993; 71: 3850–3. 10.1103/PhysRevLett.71.3850 10055089

[67] Goldman VJ, Su B, Jain JK. Detection of composite fermions by magnetic focusing. Phys Rev Lett 1994; 72: 2065–8. 10.1103/PhysRevLett.72.2065 10055779

[68] Dong J, Wang J, Ledwith PJ et al. Composite fermi liquid at zero magnetic field in twisted MoTe_2_. Phys Rev Lett 2023; 131: 136502. 10.1103/PhysRevLett.131.136502 37832017

[69] Goldman H, Reddy AP, Paul N et al. Zero-field composite Fermi liquid in twisted semiconductor bilayers. Phys Rev Lett 2023; 131: 136501. 10.1103/PhysRevLett.131.136501 37832018

[70] Foutty BA, Kometter CR, Devakul T et al. Mapping twist-tuned multiband topology in bilayer WSe_2_. Science 2024; 384: 343–7. 10.1126/science.adi4728 38669569

[71] Li W, Luhman DR, Tsui DC et al. Observation of reentrant phases induced by short-range disorder in the lowest Landau level of Al_x_Ga_1-x_As/Al_0.32_Ga_0.68_As heterostructures. Phys Rev Lett 2010; 105: 076803. 10.1103/PhysRevLett.105.076803 20868067

[72] Huang Y, Musser S, Zhu J et al. Apparent inconsistency between Streda formula and Hall conductivity in reentrant integer quantum anomalous Hall effect in twisted MoTe$_2$. Phys Rev B 2025; 112: 195136. 10.1103/ml2q-39pp

[73] An L, Pan H, Qiu WX et al. Observation of ferromagnetic phase in the second moiré band of twisted MoTe$_2$. Nat Commun 2025; 16: 5131. 10.1038/s41467-025-59691-5 40461495 PMC12134360

[74] Kang K, Qiu Y, Shen B et al. Time-reversal symmetry breaking fractional quantum spin Hall insulator in moiré MoTe$_2$ [preprint]. arXiv: 2501.02525.

[75] Li W, Redekop E, Beach CW et al. Universal magnetic phases in twisted bilayer MoTe$_2$ [preprint]. arXiv: 2507.22354. 10.1021/acs.nanolett.5c04751 41413379

[76] Knüppel P, Zhu J, Xia Y et al. Correlated states controlled by a tunable Van Hove singularity in moiré WSe$_2$ bilayers. Nat Commun 2025; 16: 1959. 10.1038/s41467-025-57235-5 40000646 PMC11861663

[77] Kang K, Qiu Y, Watanabe K et al. Double quantum spin Hall phase in moiré WSe$_2$. Nano Lett 2024; 24: 14901–07. 10.1021/acs.nanolett.4c05308 39506321

[78] Klitzing Kv, Dorda G, Pepper M. New method for high-accuracy determination of the fine-structure constant based on quantized Hall resistance. Phys Rev Lett 1980; 45: 494–7. 10.1103/PhysRevLett.45.494

[79] Thouless DJ, Kohmoto M, Nightingale MP et al. Quantized Hall conductance in a two-dimensional periodic potential. Phys Rev Lett 1982; 49: 405–8. 10.1103/PhysRevLett.49.405

[80] Niu Q, Thouless DJ, Wu YS. Quantized Hall conductance as a topological invariant. Phys Rev B 1985; 31: 3372–7. 10.1103/PhysRevB.31.3372 9936224

[81] Onoda M, Nagaosa N. Quantized anomalous Hall effect in two-dimensional ferromagnets: quantum Hall effect in metals. Phys Rev Lett 2003; 90: 206601. 10.1103/PhysRevLett.90.206601 12785910

[82] Liu CX, Qi XL, Dai X et al. Quantum anomalous Hall effect in Hg_1-y_Mn_y_Te quantum wells. Phys Rev Lett 2008; 101: 146802. 10.1103/PhysRevLett.101.146802 18851555

[83] Yu R, Zhang W, Zhang HJ et al. Quantized anomalous Hall effect in magnetic topological insulators. Science 2010; 329: 61–4. 10.1126/science.1187485 20522741

[84] Wu F, Lovorn T, Tutuc E et al. Hubbard model physics in transition metal dichalcogenide moiré bands. Phys Rev Lett 2018; 121: 026402. 10.1103/PhysRevLett.121.026402 30085734

[85] Regan EC, Wang D, Jin C et al. Mott and generalized Wigner crystal states in WSe$_2$/WS$_2$ moiré superlattices. Nature 2020; 579: 359–63. 10.1038/s41586-020-2092-4 32188951

[86] Tang Y, Li L, Li T et al. Simulation of Hubbard model physics in WSe$_2$/WS$_2$ moiré superlattices. Nature 2020; 579: 353–8. 10.1038/s41586-020-2085-3 32188950

[87] Chang X, Liu F, Xu F et al. Evidence of competing ground states between fractional Chern insulator and antiferromagnetism in moiré MoTe$_2$ [preprint]. arXiv: 2503.13213. 10.1038/s41467-026-71479-9 PMC13230869 41935041

[88] Qiu WX, Wu F. Quantum geometry probed by chiral excitonic optical response of Chern insulators. Phys Rev B 2025; 111: L121104. 10.1103/PhysRevB.111.L121104

[89] Qiu WX, Wu F. Topological magnons and domain walls in twisted bilayer MoTe$_2$. Phys Rev B 2025; 112: 085132. 10.1103/sl5k-c825

[90] Wang T, Devakul T, Zaletel MP et al. Diverse magnetic orders and quantum anomalous Hall effect in twisted bilayer MoTe$_2$ and WSe$_2$ [preprint]. arXiv: 2306.02501.

[91] Zhou WT, Dong ZY, Gu ZL et al. Itinerant topological magnons and spin excitons in twisted transition metal dichalcogenides: mapping electron topology to spin counterpart. Natl Sci Rev 2026; 13: nwaf354. 10.1093/nsr/nwaf354 PMC12878470 41660575

[92] Xiong R, Guo Y, Qin C et al. Propagating neutral modes in an intervalley coherent state [preprint]. arXiv: 2507.18770.

[93] Bernevig BA, Zhang SC. Quantum spin Hall effect. Phys Rev Lett 2006; 96: 106802. 10.1103/PhysRevLett.96.106802 16605772

[94] Sheng DN, Weng ZY, Sheng L et al. Quantum spin-Hall effect and topologically invariant Chern numbers. Phys Rev Lett 2006; 97: 036808. 10.1103/PhysRevLett.97.036808 16907533

[95] Kane CL, Mele EJ. ${Z}_{2}$ topological order and the quantum spin Hall effect. Phys Rev Lett 2005; 95: 146802. 10.1103/PhysRevLett.95.146802 16241681

[96] König M, Wiedmann S, Brüne C et al. Quantum spin Hall insulator state in HgTe quantum wells. Science 2007; 318: 766–70. 10.1126/science.1148047 17885096

[97] Knez I, Du RR, Sullivan G. Finite conductivity in mesoscopic Hall bars of inverted InAs/GaSb quantum wells. Phys Rev B 2010; 81: 201301. 10.1103/PhysRevB.81.201301

[98] Knez I, Du RR, Sullivan G. Evidence for helical edge modes in inverted InAs/GaSb quantum wells. Phys Rev Lett 2011; 107: 136603. 10.1103/PhysRevLett.107.136603 22026882

[99] Fei Z, Palomaki T, Wu S et al. Edge conduction in monolayer WTe$_2$. Nat Phys 2017; 13: 677–82. 10.1038/nphys4091

[100] Wu S, Fatemi V, Gibson QD et al. Observation of the quantum spin Hall effect up to 100 kelvin in a monolayer crystal. Science 2018; 359: 76–9. 10.1126/science.aan6003 29302010

[101] Luo XJ, Qiu WX, Wu F. Majorana zero modes in twisted transition metal dichalcogenide homobilayers. Phys Rev B 2024; 109: L041103. 10.1103/PhysRevB.109.L041103

[102] Liu X, He Y, Wang C et al. Gate-tunable antiferromagnetic Chern insulator in twisted bilayer transition metal dichalcogenides. Phys Rev Lett 2024; 132: 146401. 10.1103/PhysRevLett.132.146401 38640385

[103] Tsui DC, Stormer HL, Gossard AC. Two-dimensional magnetotransport in the extreme quantum limit. Phys Rev Lett 1982; 48: 1559–62. 10.1103/PhysRevLett.48.1559

[104] Laughlin RB. Anomalous quantum Hall effect: an incompressible quantum fluid with fractionally charged excitations. Phys Rev Lett 1983; 50: 1395–8. 10.1103/PhysRevLett.50.1395

[105] Tang E, Mei JW, Wen XG. High-temperature fractional quantum Hall states. Phys Rev Lett 2011; 106: 236802. 10.1103/PhysRevLett.106.236802 21770532

[106] Sun K, Gu Z, Katsura H et al. Nearly flatbands with nontrivial topology. Phys Rev Lett 2011; 106: 236803. 10.1103/PhysRevLett.106.236803 21770533

[107] Neupert T, Santos L, Chamon C et al. Fractional quantum Hall states at zero magnetic field. Phys Rev Lett 2011; 106: 236804. 10.1103/PhysRevLett.106.236804 21770534

[108] Regnault N, Bernevig BA. Fractional Chern insulator. Phys Rev X 2011; 1: 021014. 10.1103/PhysRevLett.110.106802 23521277

[109] Sheng DN, Gu ZC, Sun K et al. Fractional quantum Hall effect in the absence of Landau levels. Nat Commun 2011; 2: 389. 10.1038/ncomms1380 21750543 PMC3160145

[110] Bergholtz EJ, Liu Z. Topological flat band models and fractional Chern insulators. Int J Mod Phys B 2013; 27: 1330017. 10.1142/S021797921330017X

[111] Spanton EM, Zibrov AA, Zhou H et al. Observation of fractional Chern insulators in a van der Waals heterostructure. Science 2018; 360: 62–6. 10.1126/science.aan8458 29496958

[112] Xie Y, Pierce AT, Park JM et al. Fractional Chern insulators in magic-angle twisted bilayer graphene. Nature 2021; 600: 439–43. 10.1038/s41586-021-04002-3 34912084 PMC8674130

[113] Lu Z, Han T, Yao Y et al. Fractional quantum anomalous Hall effect in multilayer graphene. Nature 2024; 626: 759–64. 10.1038/s41586-023-07010-7 38383622

[114] Xie J, Huo Z, Lu X et al. Tunable fractional Chern insulators in rhombohedral graphene superlattices. Nat Mater 2025; 24: 1042. 10.1038/s41563-025-02225-7 40263582

[115] Shi J, Morales-Durán N, Khalaf E et al. Adiabatic approximation and aharonov-casher bands in twisted homobilayer transition metal dichalcogenides. Phys Rev B 2024; 110: 035130. 10.1103/PhysRevB.110.035130 38489616

[116] Ledwith PJ, Tarnopolsky G, Khalaf E et al. Fractional Chern insulator states in twisted bilayer graphene: an analytical approach. Phys Rev Res 2020; 2: 023237. 10.1103/PhysRevResearch.2.023237

[117] Wang J, Cano J, Millis AJ et al. Exact Landau level description of geometry and interaction in a flatband. Phys Rev Lett 2021; 127: 246403. 10.1103/PhysRevLett.127.246403 34951815

[118] Crépel V, Fu L. Anomalous Hall metal and fractional Chern insulator in twisted transition metal dichalcogenides. Phys Rev B 2023; 107: L201109. 10.1103/PhysRevB.107.L201109

[119] Morales-Durán N, Wang J, Schleder GR et al. Pressure-enhanced fractional Chern insulators along a magic line in moiré transition metal dichalcogenides. Phys Rev Res 2023; 5: L032022. 10.1103/PhysRevResearch.5.L032022

[120] Reddy AP, Fu L. Toward a global phase diagram of the fractional quantum anomalous Hall effect. Phys Rev B 2023; 108: 245159. 10.1103/PhysRevB.108.245159

[121] Yu J, Herzog-Arbeitman J, Wang M et al. Fractional Chern insulators versus nonmagnetic states in twisted bilayer MoTe_2_. Phys Rev B 2024; 109: 045147. 10.1103/PhysRevB.109.045147

[122] Chen J, Li Q, Wang X et al. Fractional Chern insulator and quantum anomalous Hall crystal in twisted MoTe$_2$ [preprint]. arXiv: 2504.07932. 10.1016/j.scib.2026.01.014 41620368

[123] He Y, Simon SH, Parameswaran SA. Fractional Chern insulators and competing states in a twisted MoTe$_2$ lattice model [preprint]. arXiv: 2505.06354.

[124] Li X, Chen Y, Li B et al. Deep learning sheds light on integer and fractional topological insulators [preprint]. arXiv: 2503.11756.

[125] Luo D, Zaklama T, Fu L. Solving fractional electron states in twisted MoTe$_2$ with deep neural network [preprint]. arXiv: 2503.13585.

[126] Zhang YH. Non-Abelian and Abelian descendants of a vortex spin liquid: fractional quantum spin Hall effect in twisted MoTe_2_. Phys Rev B 2024; 110: 155102. 10.1103/PhysRevB.110.155102 39303253

[127] Chou YZ, Das Sarma S. Composite helical edges from Abelian fractional topological insulators. Phys Rev B 2024; 110: 155117. 10.1103/PhysRevB.110.155117

[128] Jian CM, Cheng M, Xu C. Minimal fractional topological insulator in half-filled conjugate moiré Chern bands. Phys Rev X 2025; 15: 021063.

[129] May-Mann J, Stern A, Devakul T. Theory of half-integer fractional quantum spin Hall edges. Phys Rev B 2025; 111: L201111. 10.1103/PhysRevB.111.L201111

[130] Sodemann Villadiego I. Halperin states of particles and holes in ideal time reversal invariant pairs of Chern bands and the fractional quantum spin Hall effect in moiré MoTe_2_. Phys Rev B 2024; 110: 045114. 10.1103/PhysRevB.110.045114

[131] Abouelkomsan A, Fu L. Non-abelian spin Hall insulator. Phys Rev Res 2025; 7: 023083. 10.1103/PhysRevResearch.7.023083

[132] Wang C, Zhang XW, Liu X et al. Higher Landau-Level analogs and signatures of non-Abelian states in twisted bilayer MoTe_2_. Phys Rev Lett 2025; 134: 076503. 10.1103/PhysRevLett.134.076503 40053999

[133] Reddy AP, Paul N, Abouelkomsan A et al. Non-Abelian fractionalization in topological minibands. Phys Rev Lett 2024; 133: 166503. 10.1103/PhysRevLett.133.166503 39485960

[134] Ahn CE, Lee W, Yananose K et al. Non-abelian fractional quantum anomalous Hall states and first Landau level physics of the second moiré band of twisted bilayer MoTe_2_. Phys Rev B 2024; 110: L161109. 10.1103/PhysRevB.110.L161109

[135] Chen F, Luo WW, Zhu W et al. Robust non-Abelian even-denominator fractional Chern insulator in twisted bilayer MoTe$_2$. Nat Commun 2025; 16: 2115. 10.1038/s41467-025-57326-3 40032855 PMC11876646

[136] Shen X, Wang C, Hu X et al. Magnetorotons in moiré fractional Chern insulators [preprint]. arXiv: 2412.01211.

[137] Paul N, Abouelkomsan A, Reddy A et al. Shining light on collective modes in moiré fractional Chern insulators [preprint]. arXiv: 2502.17569. 10.1103/7sbg-yqhs 42726991

[138] Hu X, Xiao D, Ran Y. Hyperdeterminants and composite fermion states in fractional Chern insulators. Phys Rev B 2024; 109: 245125. 10.1103/PhysRevB.109.245125

[139] Girvin S, MacDonald A, Platzman P. Magneto-roton theory of collective excitations in the fractional quantum Hall effect. Phys Rev B 1986; 33: 2481. 10.1103/PhysRevB.33.2481 9938586

[140] Wolf T, Chao YC, MacDonald AH et al. Intraband collective excitations and spatial correlations in fractional Chern insulators. Phys Rev Lett 2025; 134: 116501. 10.1103/PhysRevLett.134.116501 40192353

[141] Wu F, MacDonald A. Moiré assisted fractional quantum Hall state spectroscopy. Phys Rev B 2016; 94: 241108. 10.1103/PhysRevB.94.241108

[142] Kousa BM, Morales-Durán N, Wolf TM et al. Theory of magnetoroton bands in moiré materials [preprint]. arXiv: 2502.17574. 10.1103/w57n-q4xn 41482339

[143] de Abajo FJG, Basov D, Koppens FH et al. Roadmap for photonics with 2D materials. ACS Photonics 2025; 12: 3961–4095. 10.1021/acsphotonics.5c00353 40861258 PMC12371959

[144] Xie F, Chen L, Sur S et al. Superconductivity in twisted WSe_2_ from topology-induced quantum fluctuations. Phys Rev Lett 2025; 134: 136503. 10.1103/PhysRevLett.134.136503 40250373

[145] Christos M, Bonetti PM, Scheurer MS. Approximate symmetries, insulators, and superconductivity in continuum-model description of twisted WSe$_2$. Phys Rev Lett 2025; 135: 046503. 10.1103/7z4z-vlj8 40794036

[146] Zhu J, Chou YZ, Xie M et al. Superconductivity in twisted transition metal dichalcogenide homobilayers. Phys Rev B 2025; 111: L060501. 10.1103/PhysRevB.111.L060501

[147] Kim S, Mendez-Valderrama JF, Wang X et al. Theory of correlated insulators and superconductor at $\nu$ = 1 in twisted WSe_2_. Nat Commun 2025; 16: 1701. 10.1038/s41467-025-56816-8 39962050 PMC11832926

[148] Guerci D, Kaplan D, Ingham J et al. Topological superconductivity from repulsive interactions in twisted WSe$_2$ [preprint]. arXiv: 2408.16075.

[149] Qin W, Qiu WX, Wu F. Topological chiral superconductivity mediated by intervalley antiferromagnetic fluctuations in twisted bilayer WSe$_2$. Phys Rev Lett 2025; 135: 246002. 10.1103/kf2b-r9g5 41482288

[150] Fischer A, Klebl L, Crépel V et al. Theory of intervalley-coherent AFM order and topological superconductivity in tWSe$_2$ [preprint]. arXiv: 2412.14296.

[151] Tuo C, Li MR, Wu Z et al. Theory of topological superconductivity and antiferromagnetic correlated insulators in twisted bilayer WSe$_2$. Nat Commun 2025; 16: 9525. 10.1038/s41467-025-64519-3 41152276 PMC12569151

[152] Han T, Lu Z, Hadjri Z et al. Signatures of chiral superconductivity in rhombohedral graphene. Nature 2025; 643: 654–61. 10.1038/s41586-025-09169-7 40403766

[153] Kallin C, Berlinsky J. Chiral superconductors. Rep Prog Phys 2016; 79: 054502. 10.1088/0034-4885/79/5/054502 27088452

[154] Xu C, Zou N, Peshcherenko N et al. Chiral superconductivity from spin polarized Chern band in twisted MoTe$_2$ [preprint]. arXiv: 2504.07082. 10.1103/h22z-4hsj 41557374

[155] Shi ZD, Senthil T. Doping a fractional quantum anomalous Hall insulator. Phys Rev X 2025; 15: 031069. 10.1073/pnas.2520608122 PMC12745681 41417607

[156] Zhang YH. Holon metal, charge-density-wave and chiral superconductor from doping fractional Chern insulator and SU(3)$_1$ chiral spin liquid [preprint]. arXiv: 2506.00110.

[157] Pichler F, Kuhlenkamp C, Knap M et al. Microscopic mechanism of anyon superconductivity emerging from fractional Chern insulators [preprint]. arXiv: 2506.08000. 10.1016/j.newton.2025.100340 PMC12953221 41783513

[158] Nosov PA, Han Z, Khalaf E. Anyon superconductivity and plateau transitions in doped fractional quantum anomalous Hall insulators [preprint]. arXiv: 2506.02108. 10.1103/6bgj-bfdn 41894760

[159] Shi ZD, Senthil T. Anyon delocalization transitions out of a disordered FQAH insulator [preprint]. arXiv: 2506.02128. 10.1073/pnas.2520608122 PMC12745681 41417607

[160] Chung YJ, Villegas Rosales KA, Baldwin KW et al. Ultra-high-quality two-dimensional electron systems. Nat Mater 2021; 20: 632–7. 10.1038/s41563-021-00942-3 33633355

[161] Park H, Li W, Hu C et al. Observation of high-temperature dissipationless fractional Chern insulator [preprint]. arXiv: 2503.10989.

[162] Saminadayar L, Glattli DC, Jin Y et al. Observation of the e/3 fractionally charged Laughlin quasiparticle. Phys Rev Lett 1997; 79: 2526–9. 10.1103/PhysRevLett.79.2526

[163] de Picciotto R, Reznikov M, Heiblum M et al. Direct observation of a fractional charge. Nature 1997; 389: 162–4. 10.1038/38241

[164] Papicć Z, Mong RSK, Yazdani A et al. Imaging anyons with scanning tunneling microscopy. Phys Rev X 2018; 8: 011037.

[165] Nakamura J, Liang S, Gardner GC et al. Direct observation of anyonic braiding statistics. Nat Phys 2020; 16: 931–6. 10.1038/s41567-020-1019-1 PMC8763912 35039497

[166] Bartolomei H, Kumar M, Bisognin R et al. Fractional statistics in anyon collisions. Science 2020; 368: 173–7. 10.1126/science.aaz5601 32273465

[167] Das Sarma S, Freedman M, Nayak C. Topologically protected qubits from a possible non-Abelian fractional quantum Hall state. Phys Rev Lett 2005; 94: 166802. 10.1103/PhysRevLett.94.166802 15904258

[168] Xie YM, Law KT. Orbital Fulde-Ferrell pairing state in moiré Ising superconductors. Phys Rev Lett 2023; 131: 016001. 10.1103/PhysRevLett.131.016001 37478419

[169] Zhu J, Chou YZ, Huang Y et al. In-plane magnetic field induced orbital Fulde-Ferrell-Larkin-Ovchinnikov superconductivity in twisted WSe$_2$ homobilayers. Phys Rev B 2025; 112: L020507. 10.1103/bqns-1ld3

[170] Lindner NH, Berg E, Refael G et al. Fractionalizing Majorana fermions: non-Abelian statistics on the edges of Abelian quantum Hall states. Phys Rev X 2012; 2: 041002.

